# An investigation into non-covalent functionalization of a single-walled carbon nanotube and a graphene sheet with protein G:A combined experimental and molecular dynamics study

**DOI:** 10.1038/s41598-018-37311-1

**Published:** 2019-02-04

**Authors:** Mohammad-Bagher Ebrahim-Habibi, Maryam Ghobeh, Farzaneh Aghakhani Mahyari, Hashem Rafii-Tabar, Pezhman Sasanpour

**Affiliations:** 1 Department of Medical Physics and Biomedical Engineering, School of Medicine, Shahid Beheshti University of Medical Sciences, Tehran, Iran; 2 Department of Biology, Science and Research Branch, Islamic Azad University, Tehran, Iran; 3 Department of Physics, Sharif University of Technology, Tehran, Iran

## Abstract

Investigation of non-covalent interaction of hydrophobic surfaces with the protein G (PrG) is necessary due to their frequent utilization in immunosensors and ELISA. It has been confirmed that surfaces, including carbonous-nanostructures (CNS) could orient proteins for a better activation. Herein, PrG interaction with single-walled carbon nanotube (SWCNT) and graphene (Gra) nanostructures was studied by employing experimental and MD simulation techniques. It is confirmed that the PrG could adequately interact with both SWCNT and Gra and therefore fine dispersion for them was achieved in the media. Results indicated that even though SWCNT was loaded with more content of PrG in comparison with the Gra, the adsorption of the PrG on Gra did not induce significant changes in the IgG tendency. Several orientations of the PrG were adopted in the presence of SWCNT or Gra; however, SWCNT could block the PrG-FcR. Moreover, it was confirmed that SWCNT reduced the α-helical structure content in the PrG. Reduction of α-helical structure of the PrG and improper orientation of the PrG-SWCNT could remarkably decrease the PrG tendency to the Fc of the IgG. Importantly, the Gra could appropriately orient the PrG by both exposing the PrG-FcR and also by blocking the fragment of the PrG that had tendency to interact with Fab in IgG.

## Introduction

The immobilization of oriented antibodies on nanostructures has been in the center of attraction especially in antibody-based biosensors^1^. Antibody-binding proteins, such as the PrG (protein G), supports antibody orientations in relevant immunosensors^2^. In addition, coating the substrate with the PrG can significantly increase the content of antibody loading. The PrG, selectively binds to fragment crytallisable region (Fc) of the immunoglobulin G (IgG) via key residues; Glu27, Lys28, Lys31, Gln32, Asn35, Asp40 Glu42 and Trp43 which are totally entitled fragment crytallisable receptor(FcR)^3–6^. It seems that for ordered orientation of the IgGs, these residues must face away from the surfaces. Studies have shown that the content of the loaded antigen in well-oriented antibodies is at least 3 times higher than the randomly-oriented antibodies^7,8^. Besides, it has been demonstrated that glass slides coated with the PrG could be used as a suitable substrate to orient the antibodies resulting in the detection of low concentration of antigens^9^. Furthermore, it was shown that using the PrG-coupled beads significantly improved the multiplex immunoassay approach and simultaneously quantified analytes in a limited volume^10^.

Various types of nanoparticles, including SWCNT (single-walled carbon nanotube), Gra (Graphene), and metal and magnetite nanoparticles have been mostly utilized as a scaffold for immobilization of proteins in biosensors^11–13^. SWCNT and Gra carry unique physical and chemical properties, including ultra-high charge mobility, high surface to volume ratio, and exceptional conductivity^14,15^. These properties together will result in a significant enhancement of the amount of antibody loading and electrode conductivity^16^. Therefore, employment of SWCNT and Gra leads to high selectivity, sensitivity, and stability in biosensors^17,18^. Functionalized nanostructures with the PrG have been commonly used in immunosensors^19–21^. It was confirmed that the attachment of the PrG to TiO_2_ nanoparticles considerably increased the quantity of loaded oriented antibodies in developed immunosensor^22^. Additionally, several studies have shown that when PrG maintains proper orientation and conformation, its immobilization on the surface of nanostructures could significantly improve the immunosensor activity via ordered orientation of antibodies^23,24^.

Physical adsorption of proteins on nanostructures has been rarely used due to causing protein denaturation and weak bonding, but covalent immobilization of proteins exhibits a good stability^25,26^. In the preparation process of SWCNT or Gra, their carboxylation requires a harsh oxidation step, which would damage their π-π networks, destroy their structures, and limit their emissions. Consequently, the above variations would significantly diminish their optical and electrical properties^27^. On the other hand, non-covalent immobilization of proteins on an SWCNT or Gra may not change their physical properties, thus it could be considered as an alternative^28^. Therefore, recently there have been efforts to uncover changes occurring in protein structures after non-covalent attachment of proteins with different types of nanostructures^29^. Interaction of proteins with nanostructured surfaces, based on novel binding characters, might modify the proteins structures^30,31^. It has been confirmed that non-covalent attachment of a peptide, with alpha-helical structures, to SWCNT or Gra significantly reduced its alpha-helices content^32,33^. Moreover, interaction of an SWCNT with a random coil tau protein clearly induced a beta structure^34^. Changes occurring in the proteins structures can directly affect binding of other biomolecules to the proteins and their activities^35–37^.

Although the PrG binding to functionalized gold surfaces has been thoroughly investigated, the interaction mode of this protein with other nanostructures remains unclear^38^. Furthermore, the investigation of the PrG interaction with hydrophobic surfaces is very essential^39,40^. Therefore, in the present study, experimental and molecular dynamics (MD) simulation studies have been both applied to discover the adsorption mechanism and the structural changes occurring in the PrG upon binding to an SWCNT and Gra nanostructure surfaces. First, these findings would remarkably improve the knowledge about different behaviors of similar proteins in presence of different types of CNS. Second, this study will provide a way to choose nano-sized surfaces with the least effects on the FcR fragment of the PrG structure. Finally, the interaction of the PrG with the Fc domain of mouse monoclonal IgG2b (mAb) will be investigated. Previous studies have indicated that various types of antibodies randomly interact with hydrophobic surfaces. In this regard, using functionalized hydrophobic surfaces with the PrG, which not induce structural change in its FcR, will improve antibody loading^41,42^.

## Results and Discussion

### Analysis of the PrG:SWCNT/Gra interaction and dispersion

According to the studies carried out for non-covalent adsorption of proteins on SWCNT or Gra, some parameters, including the protein hydrophobicity index^43,44^ and the number of protein aromatic residues^45,46^ have gained special attention. It has been confirmed that benzene and indole rings of aromatic amino acids (Tyr, Phe and Trp) develop strong π-π interactions with SWCNT/Gra nanomaterials^47–52^. Since protein folding may bury aromatic and hydrophobic residues, other parameters such as molecular weight of the protein should certainly be considered^43,53–55^. As the hydrophobicity index of the PrG is 34.54, and 6 aromatic residues, except histidine, exist in each domain of the PrG while holding a small and appropriate molecular weight of 31 kDa, it seemed that the PrG is a noble choice for functionalization of SWCNT/Gra^43,56^.

**Figure 1:**
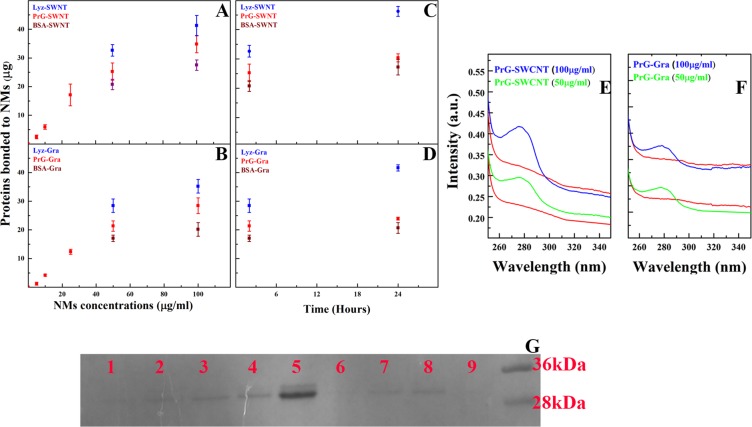
Analysis of the PrG binding to SWCNT and Gra. The Bradford protein assay results of the content of the PrG loaded on various concentrations of (**A**) SWCNT and (**B**) Gra. The amount of the PrG loaded on (**C**) SWCNT and (**D**) Gra during 24 h. Tryptophan absorbance spectroscopy was used to display amount of the PrG bound to (**E**) SWCNT and (**F**) Gra. (**G**) Comparing the amount of the PrG adsorbed on 10 µg/ml, 25 µg/ml, 50 µg/ml and 100 µg/ml of SWCNT (Lane 1–4). Lane 5, is the PrG. Lane 6–8: The PrG in presence of 25 µg/ml, 50 µg/ml and 100 µg/ml of Gra. Lane 9 is empty.

**Figure 2:**
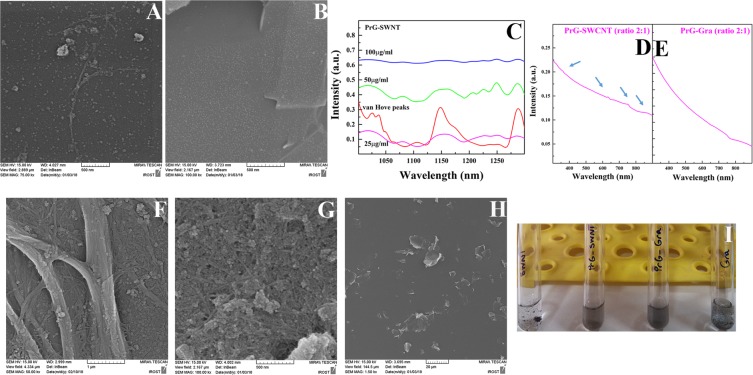
Analysis of the PrG interaction with SWCNT and Gra. SEM images of interaction of the PrG with (**A**) SWCNT and (**B**) Gra. (**C**) Optical properties of several concentrations of the PrG-SWCNT in the NIR region. Optical properties of (**D**) PrG-SWCNT and (**E**) PrG-Gra solutions (ratios 2:1) after 3 hours in the visible region. SEM images displayed dispersibility of (**F**,**G**) SWCNT and (**H**) Gra. (**I**) Photographic images of the PrG-SWCNT and the PrG-Gra dispersibility.

To confirm the interaction between the PrG and SWCNT/Gra and quantify the amount of bound PrG, different methods, including the Bradford protein assay, tryptophan absorbance, the SDS-PAGE electrophoresis, and scanning electron microscopy (SEM) were applied. Through the Bradford protein assay, the PrG concentration which has been adsorbed on nanomaterials (NMs) was indirectly quantified. As shown in Figs. 1A and B, with an increase in SWCNT/Gra concentrations from 5 µg/ml to 100 µg/ml -while keeping the concentration of the PrG constant at 100 µg/ml-, the concentration of the PrG was significantly reduced in the supernatant due to the adsorption of the PrG on SWCNT/Gra. It means that the addition of both CNS to the mixtures prepared more surfaces inducing the interaction of the PrG with both SWCNT and Gra. In order to compare the PrG interaction content with SWCNT/Gra, the other two proteins (including Lyz and the BSA) were added to the graph of the results (Figs. 1A and B). The Lyz loading content on SWCNT/Gra was significant more than the two other proteins. In addition, the PrG and the BSA adsorption content on SWCNT/Gra reached a steady state after 2 h of incubation, while the amount of Lyz adsorption increased (Figs. 1C and D). Moreover, no peaks existed around 280 nm for SWCNT or Gra alone (Figs. 1E and F). Meanwhile, after incubation of the PrG with SWCNT/Gra, a sharp peak around 280 nm was exhibited indicating that SWCNT/Gra had been functionalized by the PrG as tryptophan, showed strong absorbance around 280 nm (Figs. 1E and F). Increasing the SWCNT or Gra concentration considerably enhanced the peak demonstrating a direct relation between the SWCNT/Gra concentration and the amount of the loaded PrG. As illustrated in Fig. 1G, the PrG loading content bound to different concentrations of SWCNT/Gra was directly investigated using the SDS-PAGE electrophoresis. Apparently, by increasing the concentrations of SWCNT/Gra NMs, the amount of adsorbed PrG increased (Figs. 1G and S). Figure 1 exhibits that the PrG, like many other proteins, had more tendency to bind to SWCNT in comparison with Gra^57^. Furthermore, SEM analysis revealed that the PrG efficiently covered SWCNT/Gra surfaces (Figs. 2A and B).

Non-covalent interaction of proteins with CNS cannot individually provide sufficient hybrid biomolecules for various types of nano applications. It means that proteins must be able to disperse SWCNT/Gra in aqueous media with a relatively high yield. Studies have indicated that many proteins could cover SWCNT; however, not all of them could adequately disperse SWCNT^58^. While both cytochrome c and ferritin bounded to SWCNT, cytochrome c could satisfactorily disperse SWCNT due to high positive net charge (pI 10.8) and low molecular weight^43,59^. Some parameters including, isoelectric point of protein, protein molecular weight, ionic concentration of media, and protein: SWCNT/Gra ratio can significantly affect the dispersibility of SWCNT and Gra^43,55,60^. As the total net charge of each domain of the PrG is −4 at pH 7, its negatively charged groups mainly extend away from their SWCNT/Gra surfaces and considerably increase their dispersibility in aqueous solutions. Previous study has shown that the BSA could increase SWCNT dispersibility at pH 7^60^. In another study, Holt *et. al* have shown that Lyz with Isoelectric point of 10 and lower molecular weight could considerably provide highest yield of SWCNT dispersion in comparison with the BSA or the IgG at pH 7^55^. Therefore, the PrG with low molecular weight (nearly 31 kDa) may cover SWCNT/Gra surfaces to arrange high yield dispersibility for SWCNT/Gra.

In order to find the best ratio for the PrG-to-SWCNT/Gra to provide highest yield of dispersibility, various ratios of the PrG: SWCNT/Gra were experimentally examined. Meanwhile, the concentration of the PrG in all prepared solutions was kept constant (100 µg/ml) whereas the SWCNT/Gra concentrations varied from 5 µg/ml to 100 µg/ml. SWCNT dispersion was investigated via the NIR absorbance spectra in the range of 1000 nm to 1300 nm for analysis of the discrete van Hove peaks, 1025, 1150 and 1270 nm (shown in Fig. 2C). The reduction of absorbance along with suppression or broadening of the van Hove peaks indicated the formation of SWCNT bundles^55^. In the present study, the high quality of dispersion was carried out in the presence of 100 µg/ml of the PrG and 50 µg/ml of SWCNT (The ratio of 2:1) (Fig. 2C). These concentrations developed distinct van Hove peaks meaning that the content of SWCNT bundles was at the lowest level. When the concentration of the PrG was 4 or 10 times higher than the CNS concentrations, (4:1 and 10:1 ratios), re-aggregation of SWCNT was clearly initiated due to the free protein-mediated depletion attraction^55,61^ (Fig. 2C). As shown in Fig. 2C, when the concentration of SWCNT reached 100 µg/ml, again the content of bundles increased due to suppression or broadening of the van Hove peaks.

In order to monitor the stability of the PrG-SWCNT/Gra complexes in the solutions with the ratio of 2:1, the dispersion yield was measured over a week (Figs 1SB and 1SC)^62^. Figure 1SB illustrates that PrG-SWCNT complex in the ratio of 2:1 provides appropriate stability during a week. The content of dispersion yield in the ratio 2:1 of the PrG: SWCNT was 38 µg/ml (72%) in the beginning of the study while it reached 36 µg/ml (68%) over a week (Fig. 1SB). However, slight reduction in the absorbance at 632 nm occurred in the PrG-Gra dispersed solution indicating that the sheets of Gra started to stack during the study^63^ (Fig. 1SC). The PrG-Gra solution absorbance at 632 nm reached from 0.12219 to 0.10856 over one week of study (Fig. 1SC). Beside, optical properties in the visible region for the PrG-SWCNT and the PrG-Gra solutions were exhibited in Fig. 2D and E. Several weak peaks were highlighted for the PrG-SWCNT solution whereas no obvious peak was observed in the PrG-Gra solution (Figs. 2D and E). The SEM images have shown that the PrG: SWCNT/Gra ratios 2:1, improved the yield and the quality of SWCNT/Gra dispersion in the aqueous media (Figs. 2F–H). Moreover, photographic image of both the SWCNT and Gra after interaction with the PrG displayed their dispersibility in comparison with non-functionalized SWCNT/Gra after several weeks (Fig. 2I). In addition, the increase of zeta potential in the colloids reduces the tendency of the particles to aggregate. In order to calculate the distribution of charges on the surface of SWCNT/Gra, the zeta potential was measured. The zeta potentials of SWCNT and Gra in aqueous media were equal to −4.9 mV and −2.1 mV, respectively. The interaction of the PrG with SWCNT/Gra (PrG: SWCNT/Gra ratio 2:1) will remarkably increase the zeta potentials to −24.4 mV and −19.4 mV, respectively. Since the increment of charge distribution on the surfaces of SWCNT or Gra increased the repulsion forces, significant stability of these prepared dispersed solutions was observed. Overall, it seems that the PrG non-covalently interact with SWCNT and Gra, and based on its physicochemical properties, the PrG could also present a disperse solution with both SWCNT and Gra. Table S1 displays the proteins compared for their hydrophobicity index, molecular weight, and the SWCNT/Gra interaction.

### Analysis of secondary and tertiary structure of the PrG in presence of SWCNT/Gra

Investigating the structural changes induced in proteins upon interaction with SWCNT/Gra is a very exciting subject. It seems that these types of structural changes do not follow a similar pattern. In this regard, the final protein structure in the presence of SWCNT/Gra is greatly dependent on the protein interaction site, protein stability, and CNS shape^64^. It is exhibited that the interaction of SWCNT with both the BSA and Lyz induced partially unfolded structures by decreasing the alpha helical contents^65^. Moreover, the interaction of SWCNT with mostly-random coil Tau protein, the Bovine Fibrinogen protein, and the Gamma globulin developed a CD peak around 217 nm which is characteristic of a beta-sheet structure formation^34,44^. Furthermore, the interaction of Gra with some proteins such as telomere-binding proteins or the BSA caused a reduction in its alpha helical content^66,67^. Interestingly, no considerable structural changes were observed in some proteins interacting with Gra^68^.

**Figure 3:**
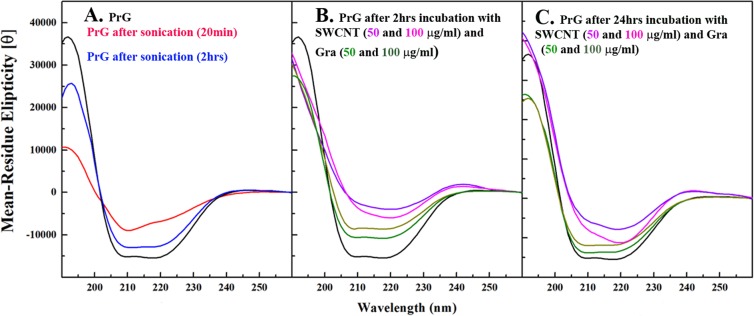
Far-UV CD spectroscopy of the PrG secondary structure under different conditions. (**A**) Far-UV CD of the PrG (200 µg/ml) and sonicated PrG alone after 20 min and 2 h. Far-UV CD measurement for the PrG in the presence of various concentrations of SWCNT and Gra after (**B**) 2 h and (**C**) 24 h. Here, sonicated PrG was added to the figure as a control.

Herein, for analysis of secondary and tertiary structures of the PrG, after sonication and upon interaction with SWCNT/Gra, Far-UV CD spectroscopy and intrinsic florescence spectroscopy were employed. Native PrG showed two negative peaks around 208 and 222 nm featuring an alpha helical structure. However, after a 20 min sonication, the significant reduction in CD measurements indicated partial changes in the secondary structure of the PrG (Fig. 3A). Based on deconvolution analysis, after sonication, the alpha helix structural content reduced from 26% to 14% and the random coil content increased from 21% to 37% (Table S2). Similar results were obtained for the BSA and Lyz that partially lost their native structure after 30 min of sonication while no significant structural changes were observed in papain and pepsin^49^. It has been reported that sonication can induce changes in secondary and tertiary structures of proteins^69^. Next, changes of the PrG secondary structure in presence of SWCNT and Gra were determined. Since the secondary structure of the sonicated PrG refolded after 2 h in the absence of SWCNT/Gra (Fig. 3A), the PrG CD characteristic and intrinsic fluorescence in presence of both CNS were determined after 2 h and 24 h. It has been demonstrated, when two negative peaks around 208 nm and 222 nm are equal, the alpha helical content is higher, whereas considerable negative enhancement in one of these CD peak intensities indicate a reduction in the alpha helix structure or the induction of parallel or anti-parallel beta structure. Interestingly, the presence of SWCNT did not allow the PrG to refold again during 2 h or 24 h of incubation (Figs. 3B and C). However, after interaction with Gra for 2 h or 24 h, the PrG refolded and its alpha helical structure was reformed again (Figs. 3B and C). Deconvolution analysis displayed obvious changes in the PrG for which the alpha helical structure content reached 11% from 26% in the presence of SWCNT (Table S2). Sharp reduction in the alpha helical structure followed with significant development of the beta and random coil structures in the presence of SWCNT after 24 h was clearly observed. Sonicated PrG in the presence of Gra refolded, and the PrG alpha helix structural content reached 28% from 26% after sonication whereas the random coil content was reduced to 25% from 37% (Table S2).

**Figure 4:**
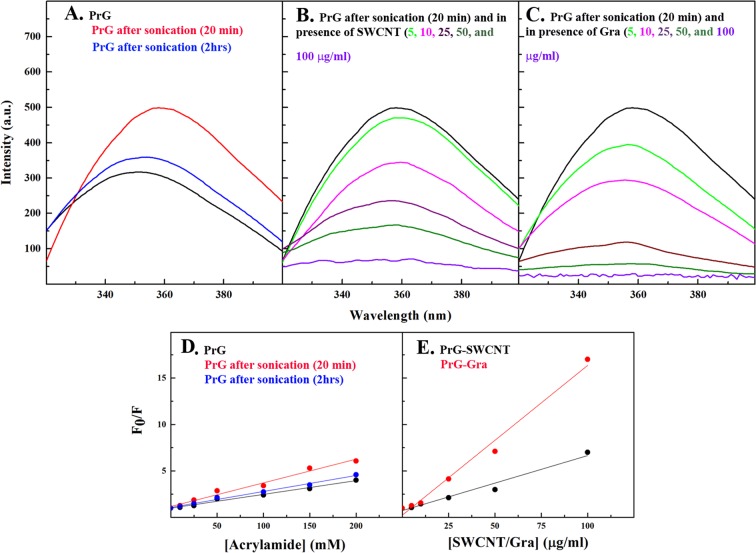
Fluorescence spectroscopy analysis of the PrG tertiary structure in the presence of SWCNT and Gra. Intrinsic fluorescence emission for (**A**) the PrG and the sonicated PrG alone after 20 min and 2 h, (**B**) the PrG-SWCNT and (**C**) the PrG-Gra. The Estern Volmer Equation was determined using various concentrations of acrylamide to calculate the Ksv values in (**D**) PrG and (**E**) sonicated the PrG. (**E**) The Estern Volmer Equation was determined using various concentrations of SWCNT or Gra to calculate Ksv values. Here, the sonicated PrG was added to the figure as a control.

Moreover, sonication caused obvious enhancement of the PrG intrinsic fluorescence accompanied by a 6-nm red shift indicating the exposure of the PrG buried hydrophobic patches to the polar solvent (Fig. 4A). Interestingly, tertiary structure of the PrG, in the absence of both SWCNT and Gra, was completely refolded (Fig. 4A). Although sonication increased the intrinsic fluorescence in the PrG, the addition of SWCNT or Gra caused to bury the hydrophobic patches again. This is determined by the sharp reduction of the intrinsic fluorescence accompanied by the slight blue shift confirming the accessibility of the hydrophobic patches in the hydrophobic environment by increasing the concentration of SWCNT or Gra (Figs. 4B and C). Enhancement of K_sv_ slope in acryl amide quenching study obviously proved the acryl amide probe locating in the proximity of the hydrophobic patches. This partially structural denaturation induced by sonication increased the PrG tendency to interact with both SWCNT and Gra (Fig. 4D). However, it seemed that Gra remarkably quenched the intrinsic fluorescence after interaction with the PrG while hydrophobic amino acids were buried in the hydrophobic environment (Fig. 4E). It has been confirmed that SWCNT or Gra sharply quench the intrinsic fluorescence of proteins through interaction with hydrophobic patches^34^. Although secondary and tertiary structures of the PrG were entirely refolded after 2 h from sonication, incubation of the PrG with SWCNT/Gra caused to induce changes in the PrG structure. Since the FcR consists of the alpha-helix and the third beta-strand, the alpha-helical and beta structural changes in the PrG, in the presence of SWCNT or Gra, may influence the PrG:IgG interactions. Therefore, the tendency of the PrG-SWCNT/Gra complexes to mAb should be investigated. Table S3 lists the secondary structural changes occurring in several proteins in the presence of SWCNT/Gra.

### Analysis of the PrG:mAb interaction

The PrG can significantly orient the IgG through its FcR. Herein, for study of the PrG-SWCNT/Gra complexes tendency to interact with the IgG, the monoclonal antibody (mAb) which includes the kappa light chain was selected. In order to define the affinity of the mAb to the PrG-SWCNT/Gra complexes, various methods, including the Bradford protein assay, the Anti-IgG_k_-HRP calorimetric assay, tryptophan absorbance, ELISA, and SEM were utilized. Before addition of the mAb to the PrG-SWCNT/Gra complexes, the BSA was added to block non-specific sites, noting that the BSA binding domain from the PrG was previously removed. Therefore, it was expected that the BSA might interact with SWCNT/Gra free spaces; however, no considerable interaction between the BSA and the PrG-SWCNT/Gra was observed (not shown).

**Figure 5:**
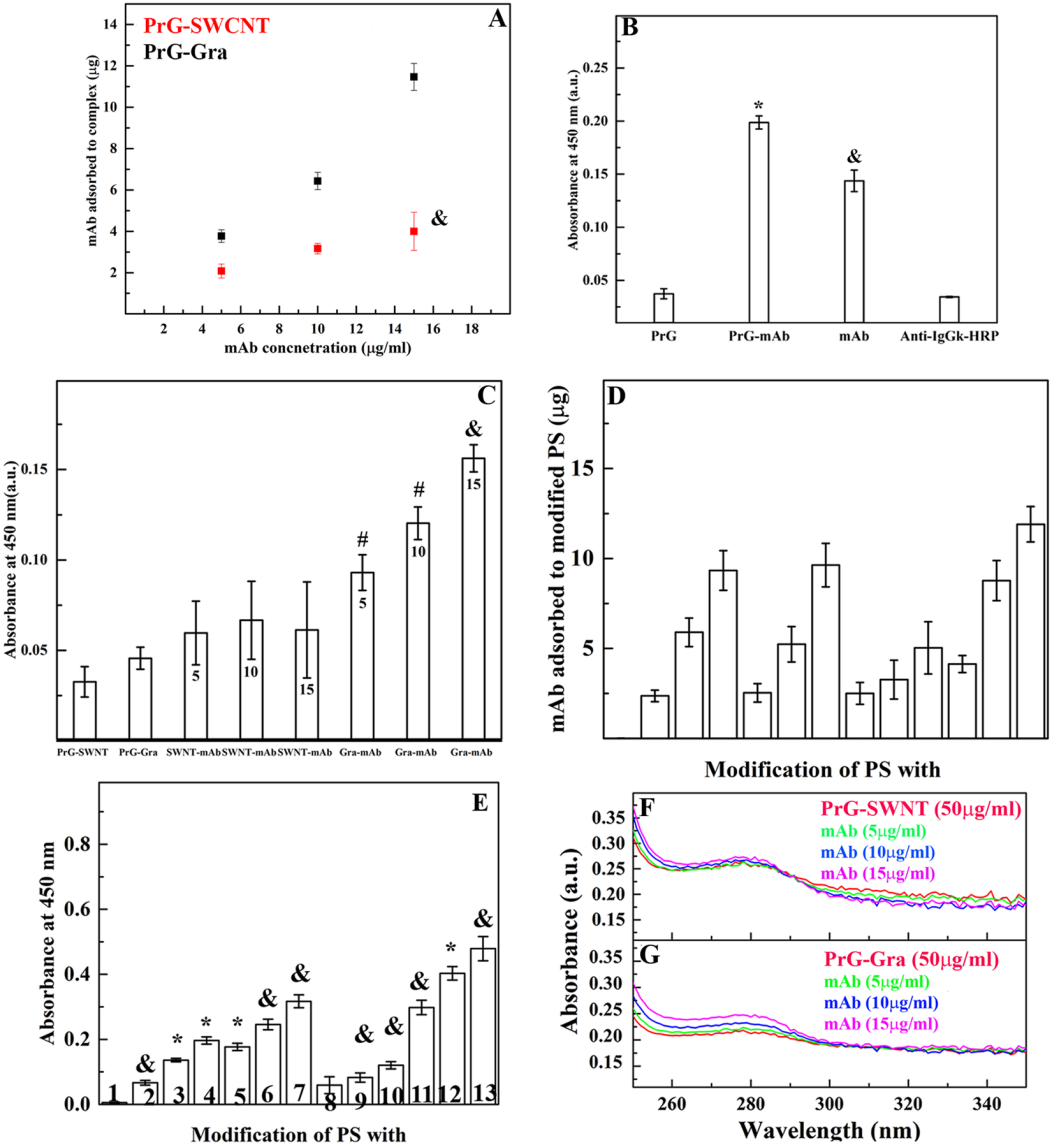
Analysis of the mAb interaction with the PrG-SWCNT/ Gra complexes. (**A**) The Bradford protein assay results of content of the mAb loaded on the PrG-SWCNT and the PrG-Gra complex. All *p* values were ^&^p < 0.01 except the one which was highlighted in the figure. (**B**) The ELISA assay for determination of the Anti-IgG_k_-HRP sensitivity for the mAb kappa light chain in presence of the PrG. (**C**) Analysis of the mAb interaction with the PrG-SWCNT/Gra and (**D**) Calorimetric assay at 450 nm was used to define the loading content of the mAb in presence of the PrG-SWCNT/Gra complex with Anti-IgG_k_-HRP. The ELISA assay for determination of amount of mAb adsorbed or well-oriented to the PS and several modified PS, including (1) PS, (2) PS-mAb (5 µg/ml), (3) PS-mAb (10 µg/ml), (4) PS-mAb (15 µg/ml), (5) PS-PrG-mAb (5 µg/ml), (6) PS-PrG-mAb (10 µg/ml), (7) PS-PrG-mAb (15 µg/ml), (8) PS- SWCNT-PrG-mAb (5 µg/ml), (9) PS-SWCNT-PrG-mAb (10 µg/ml), (10) PS-SWCNT-PrG-mAb (15 µg/ml), (11) PS-Gra-PrG-mAb (5 µg/ml) (12), PS-Gra-PrG-mAb (10 µg/ml), PS-Gra-PrG-mAb (15 µg/ml). Data are expressed as mean ± STD with n = 3. *p < 0.001; ^&^p < 0.01; and ^#^p < 0.05. UV-Visible spetra of (F) the PrG-SWCNT and (G) the PrG-Gra incubated with the three differents concentration of the mAb.

For investigation of the PrG-SWCNT/Gra complex interaction with the mAb, the ratio of 2:1 for both of the PrG-SWCNT and the PrG-Gra complexes were chosen due to their better dispersibility in this condition. First, the Bradford protein assay indirectly determined that the mAb concentration was significantly reduced in the  presence of the PrG-Gra complex in supernatant while this obvious reduction was not confirmed in the presence of the PrG-SWCNT. It was determined that in presence of SWCNT, the PrG loading was more than Gra. As SWCNT adsorbed more PrG content in comparison with Gra, we expected that the mAb was considerably loaded onto the PrG-SWCNT complex; however, its loading was remarkably reduced (Fig. 5A). Nevertheless, the PrG-Gra preserved its tendency to interact with the mAb due to the significant reduction of the PrG concentration in supernatant (Fig. 5A). Since the Anti-IgG_k_-HRP specifically binds to the kappa light chain of the mAb, after removing non-bonded mAb, the enhancement of absorbance at 450 nm indicated an interaction of the Anti-IgG_k_-HRP with the mAb bonded to the PrG-SWCNT/Gra complexes. In order to confirm that the PrG did not cover the interaction site of Anti-IgG_k_-HRP for the mAb binding, the ELISA method was utilized. No enhancement of absorbance at 450 nm was observed in the wells conjugated with the PrG alone; therefore, the Anti-IgG_k_-HRP had no tendency to interact with the PrG (Fig. 5B). However, significant absorbance was detected in the wells functionalized either with the PrG-mAb or the mAb alone. Thus, it was proved that the presence of the PrG did not occupy the kappa light chain interaction site of the mAb (Fig. 5B). No obvious enhancement of absorbance was observed in the wells modified by Anti-IgG_k_-HRP alone. Therefore, it was confirmed that the PrG increased the sensitivity of the ELISA whereas previous reports have indicated that the presence of the PrG may decrease specificity^70,71^ (Fig. 5B). Using the Anti-IgG_k_-HRP displayed that with the enhancement of the mAb concentration from 5 µg/ml to 15 µg/ml, its loading increased in the presence of the PrG-Gra, while no considerable increase was observed in presence of the PrG-SWCNT complex (Fig. 5C). Therefore, it was again confirmed that the mAb had high tendency to interact with the PrG-Gra in comparison to the PrG-SWCNT complex.

In order to compare the amount of reacted mAb with the PrG-SWCNT/Gra complexes another ELISA was performed. As illustrated in Fig. 5D, the amount of the mAb reacted with the PrG-SWCNT/Gra complexes and polystyrene (PS) alone or PS modified with the PrG were compared in ELISA. As displayed, in the Fig. 5D, modification of PS with the PrG-Gra complex could enhance the content of the mAb in three different concentrations (5, 10, 15 µg/ml). However, the content of the mAb interacted with the PrG-SWCNT was lower than its content in the PS or PS modified with the PrG alone. In the Fig. 5E orientation of the mAb was defined using interaction of Anti-IgG_k_-HRP to mAb. In the presence of the PrG-Gra complex orientation of the mAb was appropriate due to the absorbance of the PS modification with the PrG-Gra complex was two times more than modified PS with the PrG alone. It seems that modification of the PS with the PrG-Gra complex could remarkably improve both amount of the mAb loading and its orientation.

**Figure 6:**
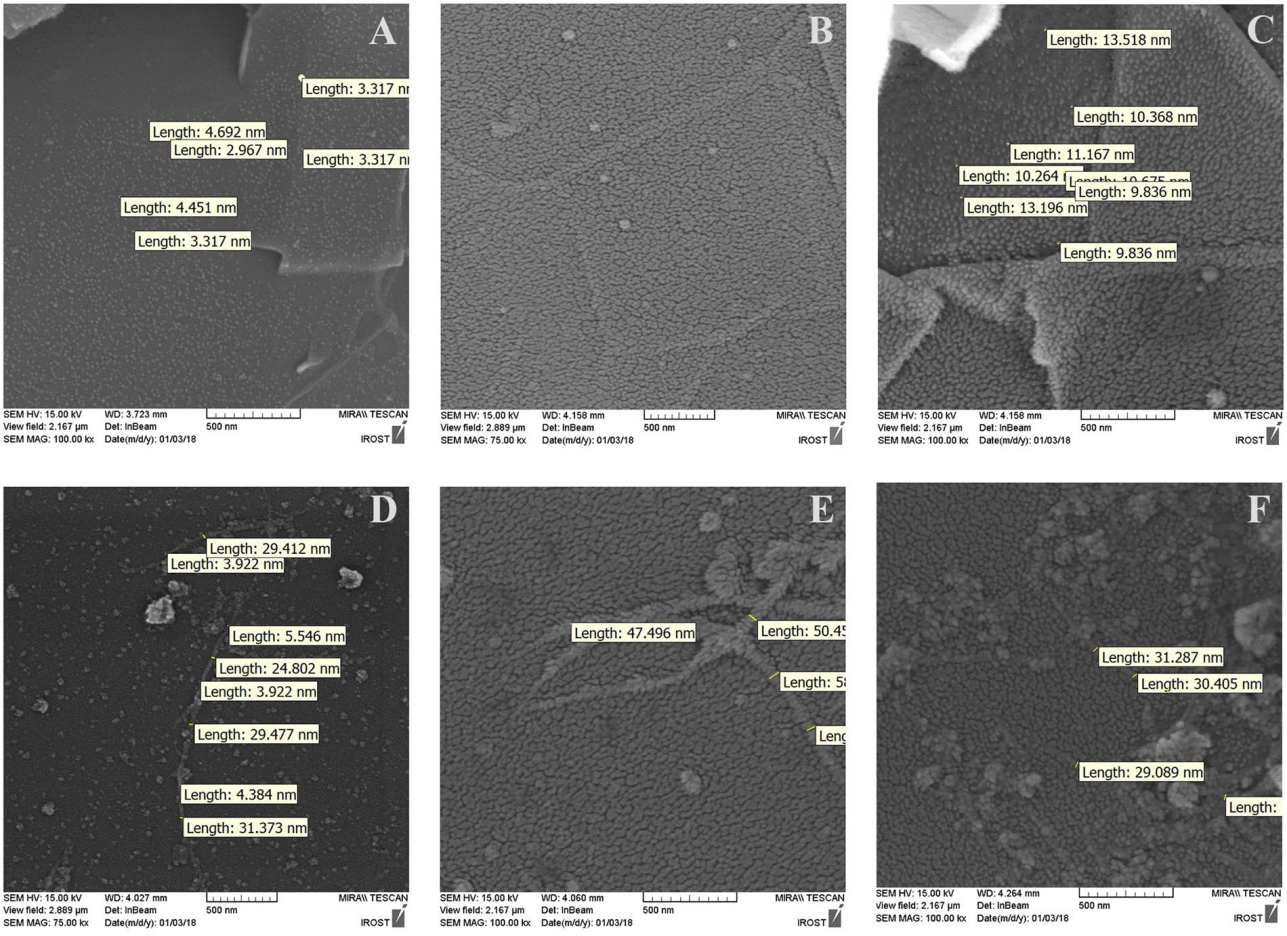
Analysis of the PrG or the mAb interaction with pristine and modified surfaces with SEM. SEM images displaying the interaction of the PrG with (**A**) Gra and (**D**) SWCNT, and interaction of the mAb with (**B**) Gra (**E**) SWCNT. The PrG-Gra (**C**) and the PrG-SWCNT (**F**) complexes were incubated with the mAb. Size of particles was determined with digimizer software based on the scale bar of images.

Moreover, Figs. 5F and G directly exhibit the mAb interaction with the PrG-SWCNT/Gra complexes with tryptophan absorbance after removing non-bonded mAb. Although the mAb was added to both the PrG-SWCNT/Gra complexes, an increase in tryptophan absorbance was not observed in the presence of the PrG-SWCNT. However, after the addition of 5, 10 and 15 µg/ml of the mAb to the PrG-Gra complex, directional enhancement of tryptophan absorbance was determined (Figs. 5F and G). Finally, interactions of the PrG with Gra and SWCNT were displayed in Figs. 6A and D. The size of the PrG on the nanostructures was around 3–5 nm. In addition, it is obvious that the final diameter of SWCNT while interacting with the PrG reached around 24 to 32 nm (Fig. 6D). Moreover, SEM images showed that when the mAb was added to both pristine Gra/SWCNT, the mAb covered Gra or SWCNT (Figs. 6B and E). Since, the mAb molecules were larger than the PrG, thus the diameter of SWCNT reached to 47 to 50 nm (Fig. 6E). In the case of Gra, the mAb interacted with the PrG-Gra complexes while larger structures were observed on the Gra surfaces and they reached to 10–13 nm (Fig. 6C). In contrast, as shown in the Fig. 6F, although the background included the mAb molecules, it was demonstrated that the mAb did not interact with the PrG-SWCNT complex. Because the diameter of the PrG-SWCNT (around 30 nm) has insignificant variation while the mAb is added to the structure (Fig. 6F)^34,44^.

Therefore, it seems that interaction of the PrG with SWCNT reduces its affinity to the IgG while no significant changes were observed in the PrG-Gra complex tendency to the IgG. Herein, the PrG was used to modify SWCNT/Gra surfaces for reasons so that increasing the loading amount of the IgG, increased SWCNT/Gra dispersibility in the media, preventing SWCNT/Gra from random interaction with the IgG. However, it is necessary to investigate both the PrG orientations adoption and structural changes that might occur in the presence of SWCNT/Gra. The FcR structure of the PrG includes the alpha helix and the third beta-strand which lie in the IgG Fc domain groove. After a structural analysis of the PrG-SWCNT/Gra complexes, it was confirmed that the adsorption of SWCNT/Gra to the PrG induced structural changes in the PrG. Based on the experimental results, it seems that the reduction in the alpha helical structure of the PrG upon interaction with SWCNT might result in the reduction of the PrG tendency for the mAb. While further molecular analysis is certainly essential to reveal the main reason occurring in the PrG in presence of SWCNT/Gra.

### Molecular dynamics simulation

In order to have a better view of the experimental results at the molecular levels, computational methods have been applied to find out molecular changes occurring in the PrG structure after the interaction with SWCNT/Gra. Due to the importance of the PrG in immunosensors manufacturing and the ELISA applications, determination of the PrG folding/unfolding as well as its orientation along the PrG interaction with surfaces, such as hydrophobic surfaces, is crucial. Thus, both docking and computational modeling studies were carried out to determine the orientations that the PrG adopts and the details of the PrG structural changes that may occur^72,73^. In this case, MD simulation is a common technique to study protein interaction with CNS^30,58,64^.

#### Investigation of major orientations that the PrG adopts in the presence of SWCNT and Gra

**Figure 7:**
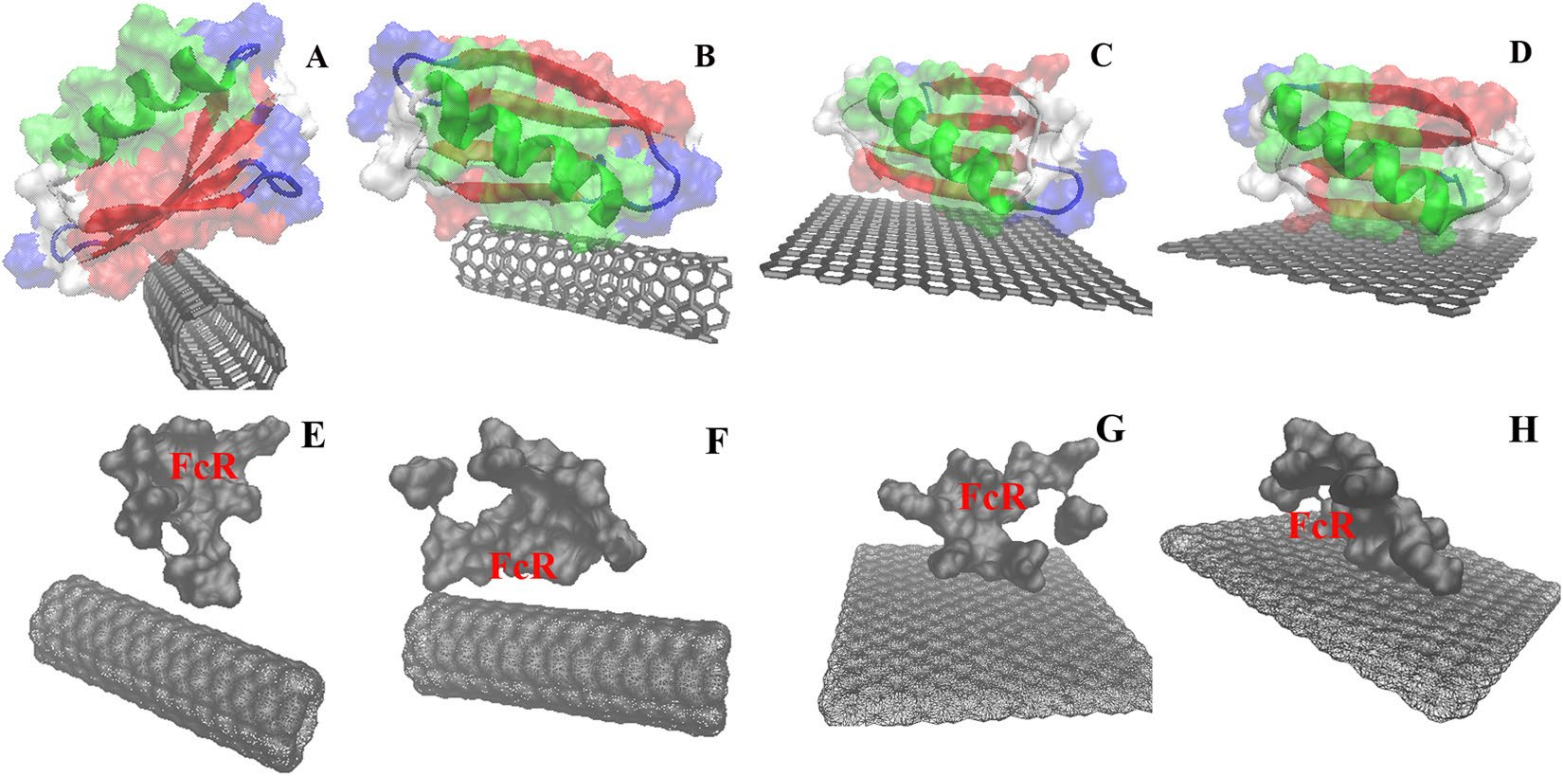
Final 2 major orientations of the PrG in the presence of (**A**,**B**) SWCNT and (**C**,**D**) Gra. The FcR of the PrG in different orientations in the presence of (**E**,**F**) SWCNT and (**G**,**H**) Gra.

In order to find the best initial orientation of the PrG in the presence of both SWCNT/Gra nanostructures for MD study, AutoDock software was used. Two major pockets were detected for each of the PrG-SWCNT and the PrG-Gra complexes (Figs. 7A–D). From these two pockets for each complex, one proper orientation was selected based on two critical reasons: holding the best free binding energy score and the highest number of clusters, and also the fact that the FcR should not be covered (Figs. 7E and G) because its coverage in the PrG with nanostructure surfaces could block the FcR:IgG interaction (Figs. 7F and H)^74^. Therefore, out of the two detected pockets, the less stable orientation, based on its free binding energy score (in which the FcR was covered), was disregarded and further MD studies were carried out on the one selected orientation for each complex. Selected conjugated structures of the PrG-SWCNT and the PrG-Gra were obtained with free binding energy values of −12.01 and −15.97 kcal/mol and cluster sizes of 58/100 and 83/100, respectively (Figs. 7A and C). Based on the number of clusters, it is more probable that SWCNT in the SWCNT-PrG complex could cover the FcR and causes significant decrease in the PrG: IgG interaction (42 from 100) (Figs. 7B and F). In contrast, through 100 PrG conformations which were generated in the presence of Gra, the FcR was rarely covered (17 from 100) (Figs. 7D and H). Therefore, it seems that the PrG adopts more appropriate orientation in the presence of Gra. Recently, Harrison and coworkers have shown that the PrG adopts two different orientations in the presence of Gra which are similar to the orientations that we have suggested in the present study (Figs. 7C and D)^39^.

In order to find the accuracy of both pockets of the PrG which were chosen in the presence of SWCNT and Gra, amino acid displacements were investigated. Adsorption of proteins with hydrophobic surfaces such as SWCNT or Gra is governed by van der Waals forces, wherein hydrophobic, π-π, and π-stacking interactions are prominent^39,75^. Previous studies indicate different affinity of amino acids to SWCNT or Gra and their affinity trends mostly include the following pattern: Aromatic amino acids > Sulphur containing amino acids > Polar amino acids, including Arg, Lys, Gln, Asn, Glu, Asp > Ser, Thr, Pro, Leu > Ile, Val and Gly^76^.

**Figure 8:**
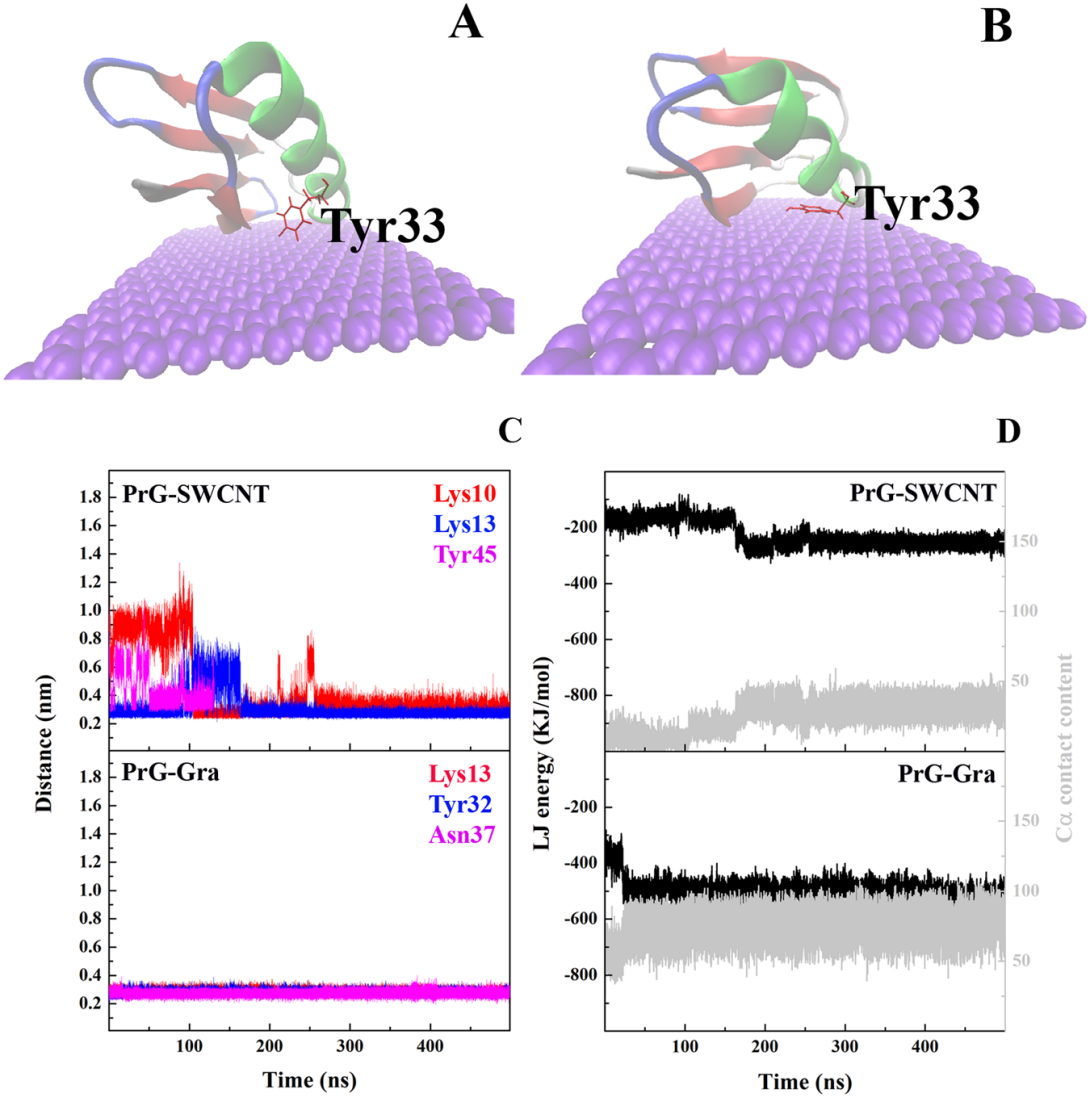
The PrG amino acids displacement. Position of Tyr 33 in (**A**) initiation and (**B**) end of MD simulation study of the PrG-Gra complex. (**C**) Minimum distances between three selected amino acids of the PrG with SWCNT and Gra during MD simulation. (**D**) vdW binding energy and the Cα content of the PrG with SWCNT and Gra during MD simulation.

As shown in Fig. S2A, the PrG binding site in the presence of SWCNT consisted of Lue6, Asn8, Lys13, Gly14 and three Threonines, including residues 44, 53, 55. However, residues involved in the PrG interaction with Gra included Thr11, Leu12, Lys13, Gly14, Glu15, Asp36 and Asn37 (Fig. S2B). During simulation, obvious amino acid displacements were observed for residues Gly9, Lys10, Thr11, and Asp46 in the presence of SWCNT, and also for residues Thr2, Thr16 to 18, Thr32 and Tyr33 in the presence of Gra. These changes in both the PrG-SWCNT/Gra complexes attempted to considerably increase their system stability with the reduction of binding energy between the PrG and SWCNT or Gra. It seems that the presence of Asn8, Lys10, Lys13, and Asp46 in the PrG-SWCNT binding site significantly reduced the PrG-SWCNT complex binding energy. Moreover, Gra induced displacement of Tyr33 in the PrG to develop π-π interactions in the PrG-Gra complex (Figs. 8A and B).

In addition, Fig. 8C displays the minimum distances between the three important Cα-residues of the PrG and carbon atoms of SWCNT or Gra during simulation. At the beginning of the simulations, minimum distances of the three major important amino acids in the PrG-SWCNT complex, including Lys10, Lys13 and Tyr45 were 0.975, 0.307 and 0.622 nm, respectively. In this regard, the minimum distance of Lys13 to SWCNT was kept constant during simulation while it was significantly reduced for Lys10 and Tyr 45 reaching 0.257 and 0.448 nm, respectively (Fig. 8C). Also, the presence of Lys13, Tyr33 and Asn37 seemed to be essential in the PrG-Gra complex development as its minimum distances were constant during simulation (Fig. 8C).

The overall, number of contacts between the Cα-residues with SWCNT or Gra significantly increased and the vdW energy in both the PrG-SWCNT and the PrG-Gra complexes was reduced (Fig. 8D). It seems that notable amino acid displacements were not observed in the pockets of the PrG in the presence of nanostructures particularly in the PrG-Gra complex, and the content of contacts and system stability simultaneously demonstrated significant increase. All this information could imply that the orientations selected for the PrG in the presence of SWCNT or Gra were appropriate. Moreover, it seems that the PrG-Gra complexes were more stable during simulation. Recently, Mont Carlo studies had predicted that the PrG may bond to Gra surfaces with two different orientations while one orientation is 15000 kJ/mol more stable than the other one^39^. The predicted stable orientation is consistent with the proposed complex of the PrG-Gra in the present study. Zuo G and co-workers showed that due to its softer character, Gra can interact with proteins easier than SWCNT or C60^64^. In addition, with a reduction in the CNS curvature, protein loading capacity was significantly increased^58^.

Herein, experimental results indicated that the amount of the PrG bound to SWCNT was more than Gra, while docking and MD simulations showed that among these selected orientations, the interaction of the PrG with Gra is energetically more stable. In addition, both SWCNT and Gra did not cover the FcR in the chosen orientations, which have a strong tendency to the Fc domain of the IgG^3^. Previous studies have confirmed that the presence of ordered layer of the IgG is applicable in immunosensors due to antigen binding efficiency^77^. It seems that Gra (not SWCNT) could enclose the PrG β-strand, which participates in the development of the PrG-Fab complex, so Gra might directly orient the PrG to interact with the Fc alone^39^.

#### Investigation of structural changes induced in the PrG in the presence of SWCNT/Gra surfaces

In addition to the PrG orientations adopted on the surface of SWCNT or Gra, structural changes induced in the PrG must be investigated. To investigate the stability of all 9 systems, the Root Mean Square Deviation (RMSD) on the Cα atoms of the PrG was calculated based on:

1 $$RMSD(t1,t2)={[\frac{1}{M}\sum _{i=1}^{N}{m}_{i}\Vert {r}_{i}({t}_{1})-{r}_{i}{({t}_{2})}^{2}\Vert ]}^{\frac{1}{2}}$$

where *M* is the number of target molecules and *r*_*i*_*(t)* is the position of molecule *i* at time *t*^78^. The RMSD values for the PrG alone and in the presence of SWCNT and Gra were calculated with respect to the initial structure of the protein. As shown in Fig. S3A, the PrG in water solution did not show sharp deviation during simulation except during the first 50 ns and from 300 to 400 ns of the simulation period. However, the presence of SWCNT in the PrG-SWCNT complex could increase the conformation deviation during simulation especially in the first 100 ns of simulation and around time courses of 150 and 350 ns (Fig. S3B). Considerable rise in the RMSD values indicated that SWCNT could induce changes in the PrG structure. On the other hand, the interaction of the PrG with Gra could not increase the RMSD values during simulation except from 400 to 450 ns just in one system which was followed by a sharp reduction (Fig. S3C). It seems that Gra could not induce any significant structural deviation in the PrG. All 9 RMSD values exhibited that the duration of 500 ns for simulation was sufficient in the present study as almost all systems were in equilibrium condition in the last 100 ns of simulation and no sharp increase in the RMSD values was observed. Since the RMSD values of the PrG in the presence of SWCNT/Gra were under 3 A°, it could imply as a confirmation of the accuracy of the selected orientations^79^. In addition, in Fig. S3, two aligned structures including alignment of the PrG with final structure of the PrG in the presence of SWCNT/Gra illustrated that the alpha helix domain diminished along simulation.

Analysis of residue fluctuations in proteins upon or after interaction with SWCNT or Gra is very crucial. Therefore, for determination of fluctuations in the PrG residues, the Root Mean Square Fluctuation (RMSF) for the PrG Cα atoms was calculated:

2 $$RMSF={[\frac{1}{T}\sum _{{t}_{j}=1}^{T}{({x}_{i}({t}_{j})-\tilde{{x}_{l}})}^{2}]}^{\frac{1}{2}}$$

where *T* is the time over which the average is taken, and x_i_ is the reference position of the particle *i*. Figures S3D, S3E and S3F display the RMSF of the PrG residues alone, and in the presence of SWCNT and Gra, respectively. Dynamics of the PrG residues in solution was in equilibrium condition except for residues from 9 to11 (Gly-Lys-Thr) and 36 to 38 (Asp-Asn-Gly) (Fig. 3SD). It is reported that the presence of amino acids with high flexibility index such as Gly > Asp > Lys > Asn > Thr significantly induces fluctuations in some parts of proteins^80^. The RMSF value of residues 9 to 11 in the PrG-SWCNT complex remarkably increased. Furthermore, attachment of the PrG to SWCNT developed significant fluctuations in residues 32 to 41, including Gln, Tyr, Ala, Asn, Asp, Asn, Gly, Val, Asp and Gly involved in the formation of the FcR (Fig. S3E). Therefore, these fluctuations may cause some changes in this essential fragment of the PrG and affect its tendency for the Fc domain of the IgG. The presence of Gra in the PrG-Gra complex considerably reduced the fluctuation of residues in the PrG (Fig. S3F). Earlier studies have shown that interaction of a peptide with proteins with NMs occasionally increased or reduced the RMSF values^81^. For example, the interaction of the BBA (mixed α/β) protein with Gra considerably increased the RMSF values. In addition, the binding of the WW domain (β-sheet) of regulatory protein with SWCNT could significantly induce structural changes and increase the RMSF values. However, neither structural changes nor the increase in the RMSF values were observed along interaction of the WW domain with Gra^81^. Interaction of the BSA with gold nano particles could remarkably induce the fluctuations in its residues^78^. In addition, the interaction of Aβ peptide with graphene oxide increased its RMSF values particularly in the C-terminal of Aβ^82^.

In order to consider the conformational diversity of the PrG in the absence or the presence of SWCNT and Gra, a clustering analysis based on the RMS deviation was performed. Result from Fig. S4A exhibits that the PrG, in the absence of SWCNT or Gra, developed transient structures while at the end of the simulation it acquired the same structure of the second cluster again. During simulation, the PrG alone appeared with 13 distinct clusters; however; the PrG in the PrG-SWCNT complex appeared with 27 distinct clusters demonstrating the fact that SWCNT could significantly induce structural changes in the PrG during the simulation period (Fig. S4B). Surprisingly, the presence of Gra in the PrG-Gra complex could not develop structural changes in the PrG (only 3 clusters appeared). On the other hand, Gra remarkably enhanced the PrG stability compared to the PrG alone (Fig. S4C).

For all systems, the DSSP (Database of Secondary Structure Assignment) algorithm was employed^83^ for assigning the secondary structure of the PrG alone and in both the PrG-SWCNT and the PrG-Gra complexes. Previously, studies were carried out to determine the effects of the CNS on protein secondary structure thorough the DSSP analysis^32,84^. Interaction of the PA_40_ (consisting of 40 alanine amino acids) with SWCNT illustrated that this entirely α-helix peptide started to lose its hydrogen bonds due to interaction. It caused the α-helix content to suddenly decrease while being converted to a turn structure^32^. Studies on lysozyme exhibited that this protein attempted to maximize its vdW interactions upon interaction with SWCNT. However, these conformational re-adjustments did not cause any apparent changes in its secondary or tertiary structures^85^. In addition, adsorption of SWCNT to alpha chymotrypsin (α-ChT) could not induce significant structural changes. It was therefore suggested that the presence of five disulphide bonds in the α-ChT considerably raised its stability in various situations, such as interaction with the CNS^85^. Here, the DSSP analysis displayed that the PrG alone, in the absence of both SWCNT and Gra surfaces, could preserve its secondary structure during simulation (Fig. S4D), meaning that aside from slight changes occurring in the PrG secondary structure, the contents of the alpha helix and the beta sheet were significantly stable. Meanwhile, in the DSSP analysis, significant structural changes occurred in the PrG in the presence of SWCNT (Fig. S4E). Presently, the alpha helical structure of the PrG in the presence of SWCNT remarkably diminished after initial 310 ns of simulation. The DSSP analysis determined that in the presence of Gra, the PrG structure did not change significantly except for slight changes occurring in some amino acids involved in the beta-sheet structures (Fig. S4F).

In addition, the number of residues in the alpha helix and the beta sheet structures was defined. Figure S5A exhibited that the number of amino acids playing roles in the alpha helix and the beta sheet structures was constant during the simulation for the PrG alone. However, the number of residues forming the alpha helix structure in the PrG-SWCNT complex was significantly reduced (Fig. S5B). In addition, slight reduction in the number of residues forming the beta sheet structure was observed in the PrG-Gra complex (Fig. S5C). At the same time, helicity length analysis determined that the PrG could preserve the alpha helix length around 2.16 nm during simulation (Fig. S6A). Meanwhile, the presence of SWCNT in the PrG-SWCNT complex reduced the alpha helical content of the PrG for which the alpha helix length in the PrG-SWCNT complex reached 1.46 nm from 2.16 nm (Fig. S6B) while its beta sheet content remained stable. Reduction of the alpha helix to bend structures in the PrG-SWCNT complex might develop alteration of the FcR pocket of the PrG. In the presence of Gra, PrG alpha helical length was constant at 2.16 nm during simulation (Fig. S6C). However, changes induced in the PrG might not affect its FcR in the alpha helical and the third beta-sheet segments. Previous study confirmed that the interaction of the PrG with Gra did not induce structural changes in the PrG^39^.

However, the present study indicated that the interaction of Gra with the PrG could not induce structural changes in the PrG alpha helix fragment while the reduction in the PrG beta sheet structure was observed. Based on the present and previous studies, it seems that structural changes occurring in proteins bonded to the CNS surfaces are significantly related to two factors: contents of the alpha helix, the beta sheet, and mixture of the α/β secondary structure, and also the interaction sites^86^.

In order to find the effects of SWCNT/Gra interactions with the PrG on the FcR pocket, distances between the FcR amino acids were obtained in the final structures of the simulations (Fig. S7). Previous studies have demonstrated that certain PrG residues, including Glu27, Lys28, Lys31, Gln32, Asn35, Asp40, Glu42 and Trp43 (FcR), which are placed within the alpha helix, the third beta strand, and its connecting loop, participate in the binding of the PrG to the Fc domain of the IgG molecules. Figures S7A and S7C demonstrate that no considerable amino acid displacements were observed in the amino acid positions involved in the FcR in both the PrG alone and in the PrG-Gra complex (triangular shape). However, remarkable changes occurred in the position of some amino acids in the FcR of the PrG-SWCNT complex by changing its arrangement to a square (Fig. S7B).

#### Calculation of the PrG affinity for the Fc domain

**Figure 9:**
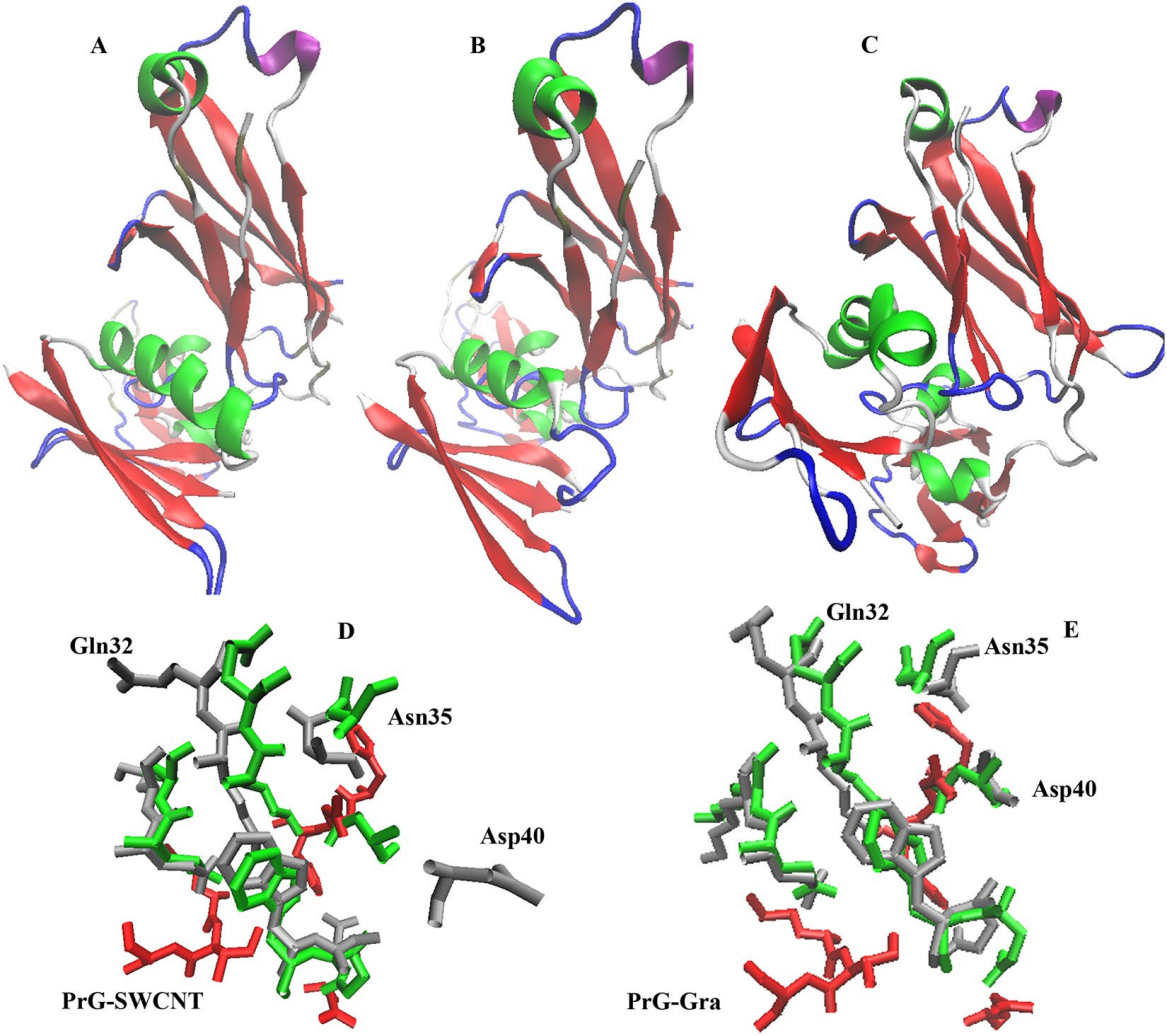
Final snapshots of Hex software. (**A**) The PrG alone. (**B**) The PrG-SWCNT and (**C**) The PrG-Gra. Amino acids displacements occurred in the FcR of the PrG while the PrG interacted with (**D**) SWCNT and (**E**) Gra. Silver = FcR amino acids for the PrG in the presence SWCNT or Gra, Green = FcR of the PrG alone, Red = amino acids in the IgG participated in interaction (Fc amino acids) with the FcR.

Significant changes occurring in the PrG FcR domain in the presence of SWCNT might result in change of its tendency for the Fc domain of the IgG. For calculation of the PrG affinity (alone and in the presence of SWCNT and Gra) for the Fc domain of the IgG, all final structures of MD simulations were extracted. Then, the docking interaction energy between the PrG and the Fc domain was calculated using the HEX 8.0.0 software. The HEX software was chosen to provide the results with high docking accuracy^87^. Herein, no obvious changes were determined in the binding energy values of the PrG and the PrG-Gra complex with the Fc domain of the IgG. Meanwhile the binding energy values of the PrG-SWCNT complex was considerably reduced and reached −8.4 kcal/mol from −56.4 kcal/mol and −49.7 kcal/mol for the PrG alone and the PrG-Gra complex, respectively. Snapshots of the PrG: IgG complexes were extracted from the Hex software (Figs. 9A–C). In addition, amino acids displacements occurred in the FcR of the PrG in the presence of SWCNT were compared to amino acid locations in the FcR of the PrG and the PrG-Gra complex. It is obvious that significant displacements occurred in Gln32, Asn35, and Asp40 of the FcR in the presence of SWCNT may cause a remarkable decrease in the system stability in the PrG-SWCNT complex in Fig. 9D and E.

## Conclusion

Presently, the interactions of the PrG with two CNSs, SWCNT and Gra, have been investigated using both experimental and MD simulation methods. First, the PrG was used to define its ability to provide ordered orientation of the IgG when directly bounded to SWCNT or Gra. It was confirmed that, due to low molecular weight, acidic pI, and amphiphilic behavior of the PrG, direct adsorption of the PrG increased SWCNT and Gra dispersibility. Then, structural changes of the PrG upon interaction with both SWCNT and Gra were well-defined using the CD spectroscopy and MD simulations. Docking studies determined two major orientations for both PrG-SWCNT/Gra complexes among which the more stable orientation for each complex did not cover the FcR. Then, MD results indicate that the interaction of the PrG with SWCNT remarkably caused to diminish its alpha helical structure. In contrast, slight structural changes were induced in the PrG in the presence of Gra, while these alterations did not display obvious changes in the FcR tendency for the Fc domain of the IgG. Finally, in contrast to Gra that thoroughly oriented PrG, it seems that neither of the orientations of the PrG adopted in the presence of SWCNT had significant affinity to interact with the IgG; the first orientation revealed diminished alpha helical structure and the second one demonstrated coverage of FcR.

## Materials and Methods

### Materials

SWCNT (755710), Gra (806668), PrG (l9459) and Bovine Serum Albumin (BSA), Lysozyme (Lyz), 2,2′-Azino-bis (3-ethylbenzothiazoline-6-sulfonic acid) diammonium salt (ABTS-A1888), hydrogen peroxide 30 wt.% in H_2_O (216763), Coomassie Brilliant Blue G-250 (1.15444) were purchased from Sigma-Aldrich chemical company (St. Louis, MO, USA). Mouse monoclonal IgG2b (mAb) (sc-69887) and mouse IgGk binding protein-HRP (Anti-IgGk-HRP) (sc-516102) were both purchased from Santa Cruz Biotechnology Inc, USA. We used Pierce Antibody clean-up kit (44600) (Thermo Fisher Scientific company, USA) to remove protein stabilizers. All other chemicals were analytical grade and were from Merk.

### SWCNT and Gra dispersion

SWCNTs and Gra were washed three times with ethanol to remove amorphous carbon structures (Centrifuge 5804 R, Eppendorf, Germany). After getting dried, in order to remove aggregate structures, SWCNTs and Gra were dispersed in dimethylformamide (DMF) using a sonicator (Elmasonic –Elma schmidbauer GmbH, Germany) followed by centrifugation (1250 g, 10 min). This step of centrifugation (1250 g, 30 min) was repeated until no cluster or aggregate structures were settled down in that speed. When the supernatant of the solution got dried, a 2 mg/ml of SWCNTs or Gra was dispersed in DMF for 30 min in ice cool box. Then from the prepared stock solutions, various concentrations of SWCNT and Gra, including 5, 10, 25, 50 and 100 µg/ml were made in the PBS (10 mM, pH 7). Afterward, diluted solutions were again sonicated with probe No. 3 (ultrasonic cell crusher, Ningbo HaishuSklon Electronic Instrument Co. Ltd, China) for 6 min so that all SWCNT and Gra structures were de-bundled (10 s run/10 s stop). Fresh sonicated PrG/BSA/Lyz (stock solution of 2 mg/ml for 2 min 10 s run/10 s stop in bath sonicator) was added to the prepared SWCNT/Gra mixtures (at final concentration of 100 µg/ml). Then, all mixtures were again dispersed in an ice bath sonicator for 20 min (Frequency 37 kHz and 25 °C). Finally, all prepared solutions and different concentrations of SWCNT and Gra were incubated overnight on rotor at 800 RPM and 25 °C (Thermomixer comfort, Eppendorf, Germany)^88,89^.

### Analysis of protein-NMs interactions

Interaction of proteins with the CNS was studied with different techniques. In order to determine the concentrations of the PrG loaded on an SWCNT and Gra, the Bradford method was utilized using the BSA (at concentrations of 2.0 to 20.0 µg/ml) as a standard. After sufficient incubation time of SWCNT, Gra and proteins, all samples were centrifuged at 22000 g for 30 min, and the colorless supernatant was collected. Then, the concentration of the PrG in the supernatant of the samples was determined and subtracted from the initial concentration of protein^90^. Briefly, for the Bradford method, 50 µL of samples (collected supernatant) was added to 950 µL of reagent (100 mg Coomassie Brilliant Blue G-250 in 50 ml 95% ethanol to which 100 ml of 85% (w/v) phosphoric acid was added per liter). The solutions absorbance was read (the UV-VIS spectrophotometer UV mini1240 CE Shimadzu, Japan) at 595 nm after 5 min of incubation at 25 °C^91^. Moreover, the SDS-PAGE electrophoresis (15% acrylamide) was employed to display the PrG binding to SWCNT/Gra. All prepared gels were stained with Coomassie blue. The electrophoresis was justified with the PageRulerTM pre-stained protein ladder, 10 to 180 kDa, Thermo Scientific company, USA (26616)^91^. Additionally, the UV-visible spectra of the CNS (50 and 100 µg/ml) in the presence of the PrG (100 µg/ml) were determined using the NanoDrop One (Thermo Scientific, USA)^90^. In order to prepare the sample for analysis of the PrG-SWCNT/Gra adsorption or to study structural changes occurred in the PrG in the presence of SWCNT/Gra nanostructures, non-bonded PrGs were removed from the solution by washing the samples with 10 mM PBS, pH 7 (centrifuged at 22000 g for 30 min for three times). Furthermore, the zeta potential of the PrG-SWCNT/Gra was measured using the Zeta sizer nano-ZS (Malvern Institute, Malvern, UK). Optical properties of SWCNT/Gra in the presence of the PrG were determined in the Vis-NIR region. Then, the dispersion yield of the prepared solution in the ratio of 2:1 was measured^63^. The yield of dispersion was calculated based on the equation 3

3 $$\frac{[SWCNT]\,at\,632\,nm}{[SWCNT]\,initial}= \% \,dispersion\,yield$$

### Scanning Electron Microscopy (SEM)

Twenty µl of samples were placed on the center of small glasses washed for three times with pure ethanol. After drying, the samples were deposited with thin layer of gold. Finally, all samples were observed with field emission SEM (MIRA II LMU, TESCAN, Czech Republic).

### Circular Dichroism Measurement

The Aviv Model 215 Spectropolarimeter (Lakewood, New Jersey, USA) was used to investigate the secondary structure of the PrG employing a spectral window of 190–260 nm (Far-UV). All scans with a time constant of 2 s and scan rate of 10 nm/min in quartz cells, with length of 1 mm light path, were recorded at room temperature. All the PrG concentrations for the Far-UV CD measurements were 200 µg/ml. In addition, the mean residue ellipticity [*θ*] was calculated using the equation

4 $$[\theta ]=(\frac{\theta obs}{10})MRW/CL$$

where *θ*_*obs*_ is the observed ellipticity in degrees, MRW is the mean residue molecular mass (31 kDa), *C* is the PrG concentration in g/ml, and *L* is the path length in cm. All final spectra were extracted after subtracting the SWCNT or Gra graphs which were smoothed over^92^. Afterward, all graphs were analyzed via deconvolution analysis using the CDNN software.

### Fluorescence Spectroscopy Measurement

In order to measure both the intrinsic fluorescence and the acryl amide quenching of the PrG (final concentration of 100 µg/ml) in the presence of different concentrations of SWCNT and Gra, the Carry Eclipse (Varian, Australia) spectroflourometer was used. For both analysis, the PrG was excited at 280 nm and the emission was recorded at 330–400 nm region. Emission and excitation slits were set to 5 and 10 nm, respectively. Moreover, the Stern-Volmer equation was utilized to calculate the fluorescence quenching of the PrG in the presence of acrylamide for comparison of the native and sonicated PrG, and the interaction of the PrG with SWCNT/Gra. Ksv values were calculated from the slope of the Stern-Volmer graph (equations 5 and 6)^34,93^:

5 $$\frac{F0}{F}=Ksv\,[Acrylamide]+1$$

6 $$\frac{F0}{F}=Ksv[SWCNT\,or\,Gra]+1$$

### Comparing loaded content of the mAb in the presence of the PrG-SWCNT/Gra complex

In order to determine the tendency of the mAb to both the PrG-SWCNT/Gra complexes, nonspecific sites of both complex surfaces were blocked first with a solution of 5% BSA (1 hour, RPM 800, 25 °C) and the non-bonded BSA were removed. Next, the mAb (final concentrations of 5, 10 and 15 µg /ml) was added to the prepared samples and incubated for 1 h (800 RPM, 25 °C). Accordingly, to remove the excess mAb, samples were centrifuged three times at 22000 g for 30 min with the PBS buffer (10 mM, pH 7). The Bradford protein assay (calibrated with concentrations of 2.0 to 20.0 µg/ml of the mAb), and UV-visible spectroscopy and SEM were employed for investigation of the mAb loaded content. In addition, calorimetric assay (explained below) using the Anti-IgGk-HRP was applied after removing the non-bonded Anti-IgGk-HRP to define the content of the mAb loaded on the PrG-SWCNT/Gra complex.

### Quantification of the mAb with the Anti-IgG_k_-HRP in ELISA

For quantification of the mAb with the Anti-IgG_k_-HRP in the presence of the PrG, the Enzyme-Linked Immunosorbent Assay (ELISA) was utilized. First, microtiter ELISA plates (Tissue Culture Testplate, SPL Life Science Ltd. Korea) were coated with 100 µl PrG (10 µg/ml), in coating buffer (100 mM NaHCO3, pH 9.6), for 2 h at 37 °C. Then, the coating solution was removed and the plate was washed three times with 200 µl of the PBS (10 mM Na_2_HPO_4_, 1.8 mM Na_2_H_2_PO_4_, and 0.2%Tween 20, pH 7.4) for 5 min. Uncoated sites were blocked with 100 µl of blocking buffer (5% BSA in 100 mM NaHCO_3_, pH 9.6) for 1 h at 37 °C. After removing the blocking buffer and washing the plates for three times with washing buffer, 100 µl of the mAb (10 µg/ml in coating buffer) was added to the plates and incubated for 2 h at 37 °C. The non-bonded mAb was removed from the plates by washing three times. 100 µl of 1:1000 (400 ng/ml) and 1:10000 (40 ng/ml) of the Anti-IgG_k_-HRP (in coating buffer) was then added and the mixture was incubated at 37 °C for 2 h. Finally, after washing and removing the non-bonded Anti-IgGk-HRP using the washing buffer, the enzymatic reaction was developed using the ABTS as the substrate. After 30 min, 100 µl from 2 M acid sulphuric solution was added to inhibit the reaction. The absorbance at 450 nm was measured with an ELISA plate reader (HALO LED 96, Dynamica. Scientific Ltd. Korea). Each experiment was repeated three times^94^. In order to determine the effects of polystyrene (PS) ELISA plates modification with the PrG-SWCNT or the PrG-Gra, 100 µl of the PrG-SWCNT and the PrG-Gra (2:1 PrG: SWCNT/Gra) complexes were added to the PS plates. After 2 h incubation at 37 °C, the plates were washed three times with 200 µl of the PBS for 5 min to remove nonbounded complexes. Then other steps followed as mentioned above. In addition, three different concentrations including (5, 10 and 15 µg/ml) of mAb was used for determination of the mAb affinity to the PrG-SWCNT, PrG-Gra, and the PrG-PS surfaces. The amount of unreacted antibody in the supernatant was measured using the Bradford assay method, to indirectly determine the amount of the mAb interacted to modified surfaces. Herein, the absorbance at 450 nm was measured using the NanoDrop.

### Molecular docking studies

The initial three dimensional structures of the PrG and the PrG-Fc domain complex were obtained from the Protein Data Bank with the PDB codes for 1PGB and 1FCC, respectively. Three dimensional coordinates of both nanostructures, the SWCNT and Gra, were built by the Visual Molecular Dynamics 1.9.2 (VMD) software^95^ (Table S4). Molecular rigid docking calculations of the PrG-SWCNT/Gra were performed by AutoDock 4.2 software^96^ to energetically predict the best sites for the nanostructure protein attachment. Essential hydrogens and charges were added to the protein and both nanostructure charges were kept zero. Both nanostructure active bonds were considered to be non-rotatable. The entire protein was centered and fixed in a grid box and the calculations were performed via the Lamarckian Genetic Algorithm^97^. For further analysis, the Auto Dock tools 1.5.4 (ADT)^98^ and the VMD software were both employed. Three docking studies were carried out for each of the PrG-SWCNT and the PrG-Gra complexes and 100 structures were generated in each docking, among which two major pockets were detected for each complex. From these two pockets for each complex, one proper orientation was selected and further MD studies were carried out on the one selected orientation for each complex.

### MD simulation of the docked structure

Final structures obtained from the PrG-SWCNT/Gra dockings were used as the initial PDB structures for MD simulations. All simulation studies were performed using the GROMACS package (version 4.5.5)^99^ using high performance clusters. Optimized potentials for liquid simulations all-atom (OPLS-AA) force field^100–104^ were employed to parameterize the protein and the non-bonded interactions between the PrG and the two nanostructures, SWCNT and Gra, in all simulations^105–107^. In this study, SWCNT or Gra carbon atoms types were defined as naphthalene, tryptophan (TRP), and tyrosine (TYR) and the corresponding bond and angle types were defined as phenylalanine (PHE). Moreover, the dihedral angle type was parameterized as an aromatic ring property in the OPLS-AA force field. The van der Waals (vdW) interactions between carbon atoms were modeled according to sp^2^ carbons in OPLS-AA force field. The equilibrium distance and the well depth were 3.55 A° and 0.29288 KJ/mol, respectively^101,102,108^.

To determine the effects of the chosen nanostructures on the PrG in a water environment, all MD simulations were performed in a TIP3P model of water molecules in a cubic box. Since the total net charge of the PrG in pH 7 was −4, four Na+ ions were replaced with water molecules in the systems. Periodic boundary conditions (PBC) were applied in all three dimensions. For long-range electrostatic interactions, the Particle Mesh Ewald method was utilized and cutoff distances of 1.0 nm were selected for electrostatic and vdW interactions^109^. All solute bonds and water geometry were both constrained using the LINCS and SETTLE algorithms^110^.

To trap the initial structures in local minima of the potential energy landscape, energy minimization with the steepest descent algorithm was carried out on the whole system until the maximum force was smaller than 10 kJ/mol. Afterward, two separate MD simulations were sequentially carried out for adjusting the temperature and equilibration of the solvent, ions around the protein, and the nanostructures while the protein backbone and nanostructures were simultaneously restrained. In the first step, a constant- (NVT) MD simulation was performed for 100 ps to adjust the temperature of the system at 300 K using velocity rescaling (υ-rescale) thermostat method when tau-t was 0.1 ps^111,112^. It was then followed by a constant-(NPT) MD simulation for 100 ps to control the pressure of the system at 1 bar using the Parrinello-Rahman algorithm when tau-p was set equal to 2.0 ps^113^. Before starting the final MD simulations, another MD simulation was run for 100 ps while the nanostructures were still restrained to stabilize the system again. Final MD simulation time step was set at 2.0 fs and the coordinates information was registered every 20 ps. The SWCNT and Gra were fixed throughout the final MD simulations.

Nine independent MD simulations were performed, with three simulations for each complex and three simulations for the protein alone as the system control. Each simulation was run for 500 ns. The total simulation time was 4.5 microsecond. All analyses were performed on the centered and fitted XTC file obtained at the end of the MD simulation studies.

### Hex software

After 500 ns of simulation, all final structures of the PrG in absence and presence of both nanostructures, the SWCNT and Gra, were used to calculate their binding energy values with the Fc domain of the IgG. Since two complexes of the PrG-Fc domain existed in the 1FCC pdb file, one complex was selected and the other one was removed from the file. The modified 1FCC pdb file was opened into the software and binding sites were introduced for the software. Afterward, both nanostructures were removed from the final structures obtained from the MD simulations. Then, all data in the PrG files were changed to the data in the modified 1FCC file. Here, the PrG as a ligand and the Fc domain as a receptor were opened in the HEX 8.0.0 software and the docking calculation was initiated^114^. Since each situation was simulated three times, for all the 9 PrG files, the HEX calculation was performed and the final mean energy values for each of three different situations are reported.

### Statistical Analysis

All experimental calculations were repeated three times. The results are displayed as mean ± standard deviation and a student’s paired t-test was employed to analyze the statistical significance. *P* values less than 0.001, 0.01 and 0.05 were taken as statistically significance: *p < 0.001; ^&^p < 0.01; and ^#^p < 0.05.

## Supplementary information

Supplementary Material.
