# Supplementary material for: An investigation into non-covalent functionalization of a single-walled carbon nanotube and a graphene sheet with protein G:A combined experimental and molecular dynamics study
Source: Sci Rep. 2019 Feb 4;9:1273. doi: 10.1038/s41598-018-37311-1 (PMC6362288; doi:10.1038/s41598-018-37311-1)
Supplement: Supplementary file 1 — Supplementary Material. [file 41598_2018_37311_MOESM1_ESM.docx]

**An investigation into non-covalent functionalization of a single-walled carbon nanotube and a graphene sheet with protein G:A combined experimental and molecular dynamics study**

**Mohammad-Bagher Ebrahim-Habibi^1^, Maryam Ghobeh^2^, Farzaneh Aghakhani Mahyari^3^, Hashem Rafii-Tabar^1^, Pezhman Sasanpour^1*^**

**^1^**Department of Medical Physics and Biomedical Engineering, School of Medicine, Shahid Beheshti University of Medical Sciences, Tehran, Iran.

**^2^** Department of Biology, Science and Research Branch, Islamic Azad University, Tehran, Iran.

**^3^**Department of Physics, Sharif University of Technology, Tehran, Iran.

* Corresponding Authors: PS: [pesasanpour@sbmu.ac.ir](mailto:pesasanpour@sbmu.ac.ir) HRT: [rafii-tabar@nano.ipm.ac.ir](mailto:rafii-tabar@nano.ipm.ac.ir)

**Supplementary Data**

**Figure S1.**

**
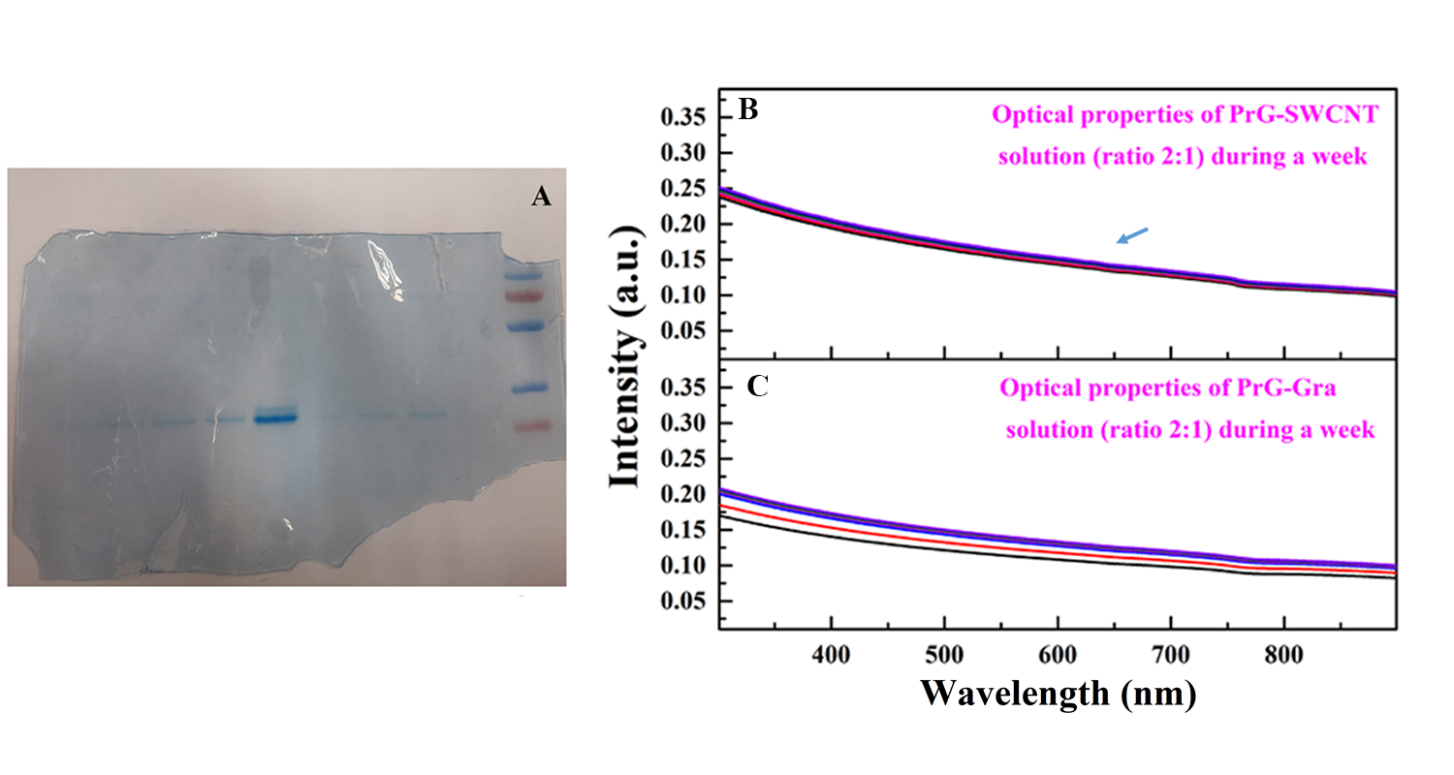
**

**Figure S1.**(A) Comparing the amount of the PrG adsorbed on 10µg/ml, 25µg/ml, 50µg/ml and 100µg/ml of SWCNT (Lane 1-4). Lane 5, is the PrG. Lane 6-8: the PrG in presence of 25µg/ml, 50µg/ml and 100µg/ml of Gra. Lane 9 is empty. Visible region spectra for (B) PrG-SWCNT and (C) PrG-Gra solutions, ratio 2:1 over a week.

**Figure S2.**

**
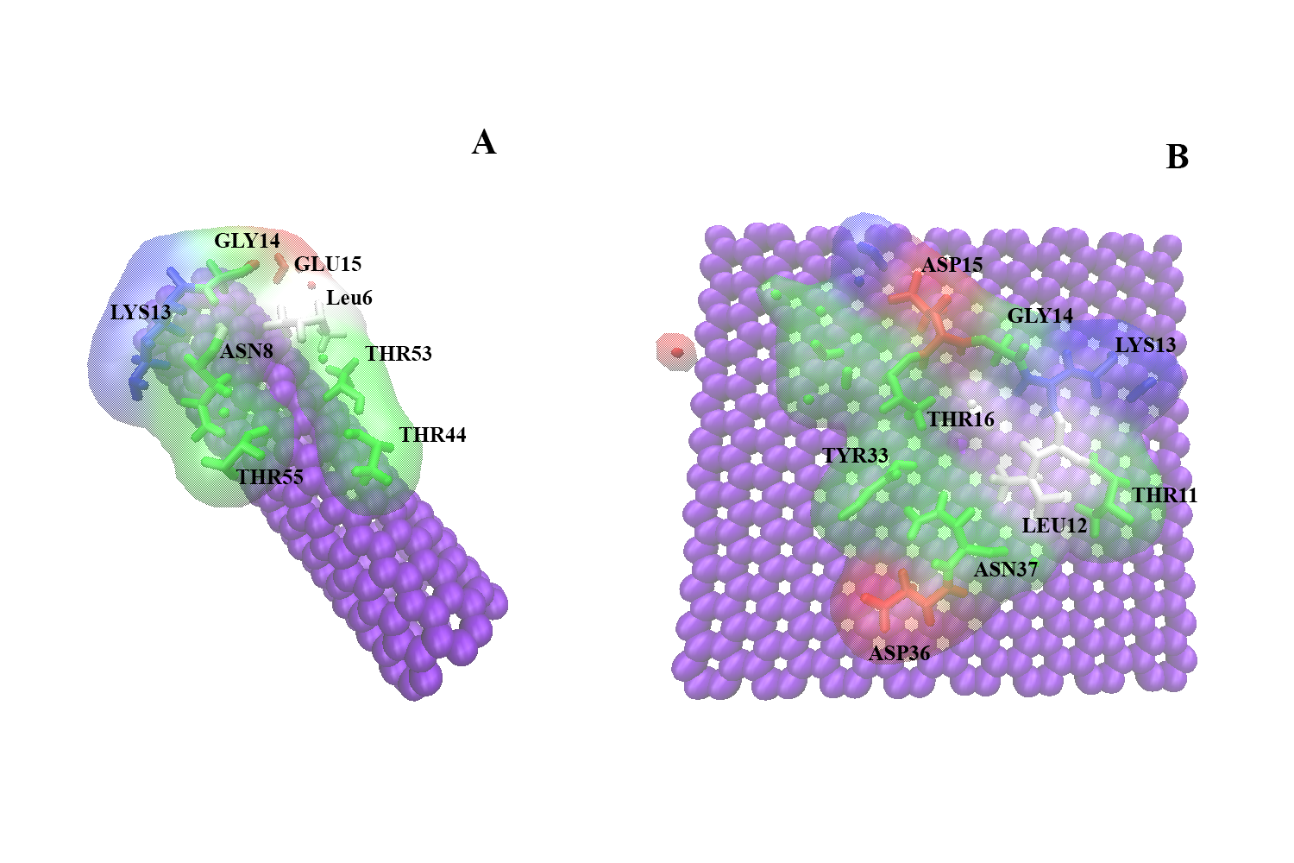
**

**Figure S2.** PrG binding site with (A) SWCNT and (B) Gra (all amino acids are located under 5 A˚ from SWCNT and Gra).

**Figure S3.**

**
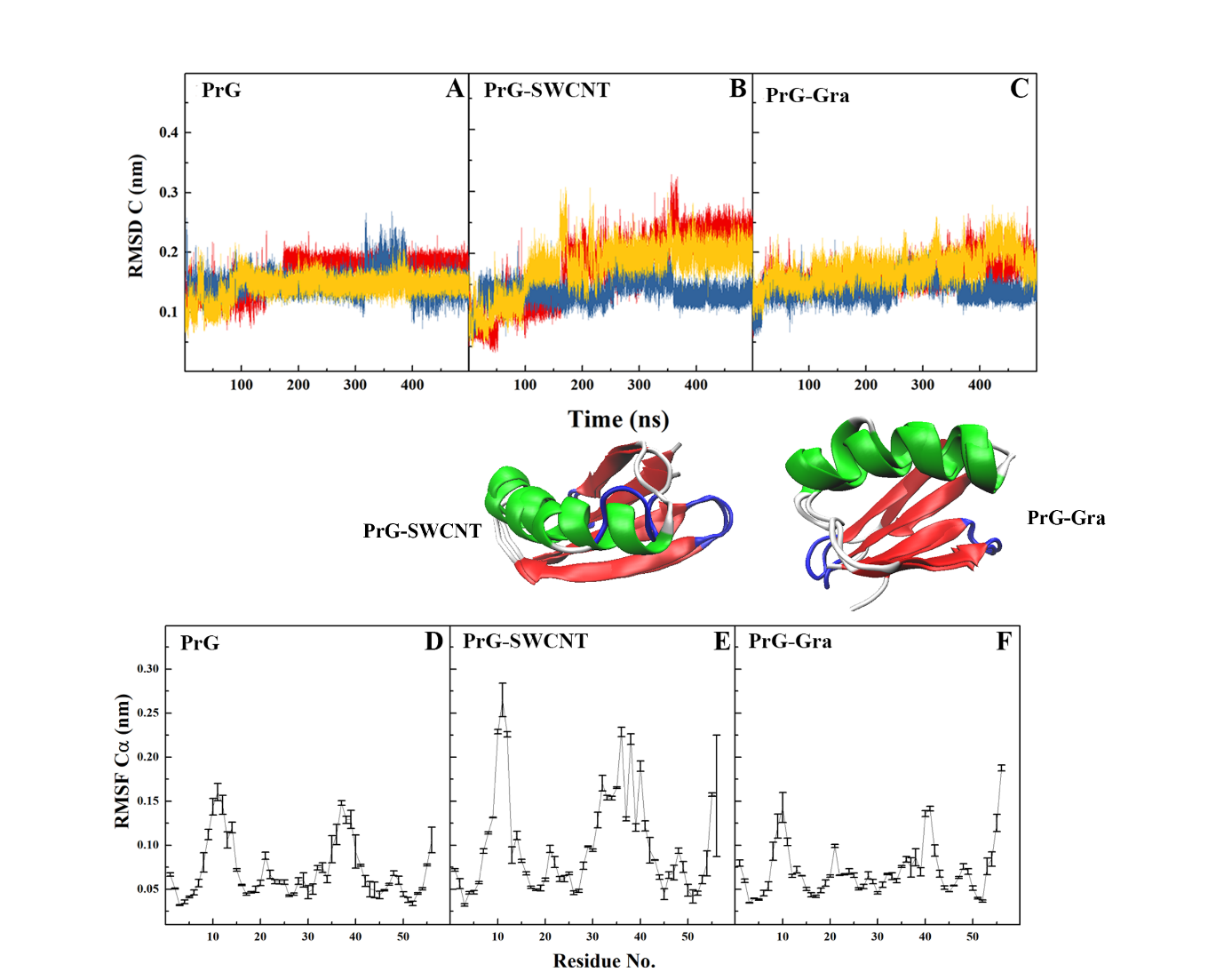
**

**Figure S3.** Cα RMSD and Cα RMSF values for the PrG-SWCNT/Gra during 500 ns simulation study. (A) Cα RMSD values for (A) the PrG alone, (B) the PrG-SWCNT complex, and (C) the PrG-Gra complex. Cα RMSF values for (D) the PrG alone, (E) the PrG-SWCNT complex, and (F) the PrG-Gra complex. PrG with final PrG in presence of SWCNT and final PrG in presence of Gra were aligned.

**Figure S4.**

**
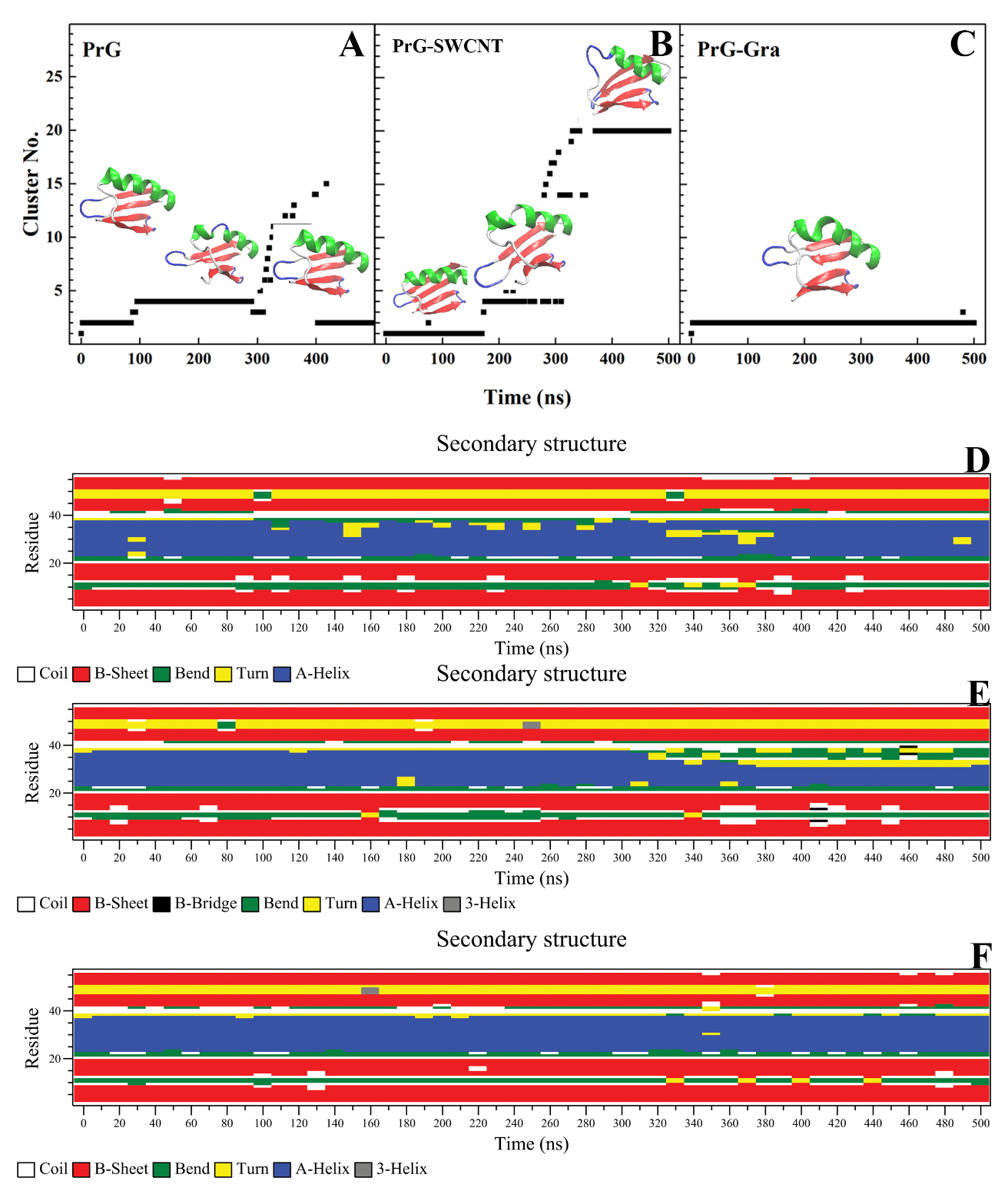
**

**Figure S4.** Structural analysis of the PrG in absence and presence of SWCNT/Gra. (A) Cluster analysis of the PrG alone. (B) Cluster calculation of the PrG-SWCNT and (C) cluster calculation of the PrG-Gra. DSSP analysis for (D) the PrG alone, (E) the PrG-SWCNT complex, and (F) the PrG-Gra complex.

**Figure S5.**


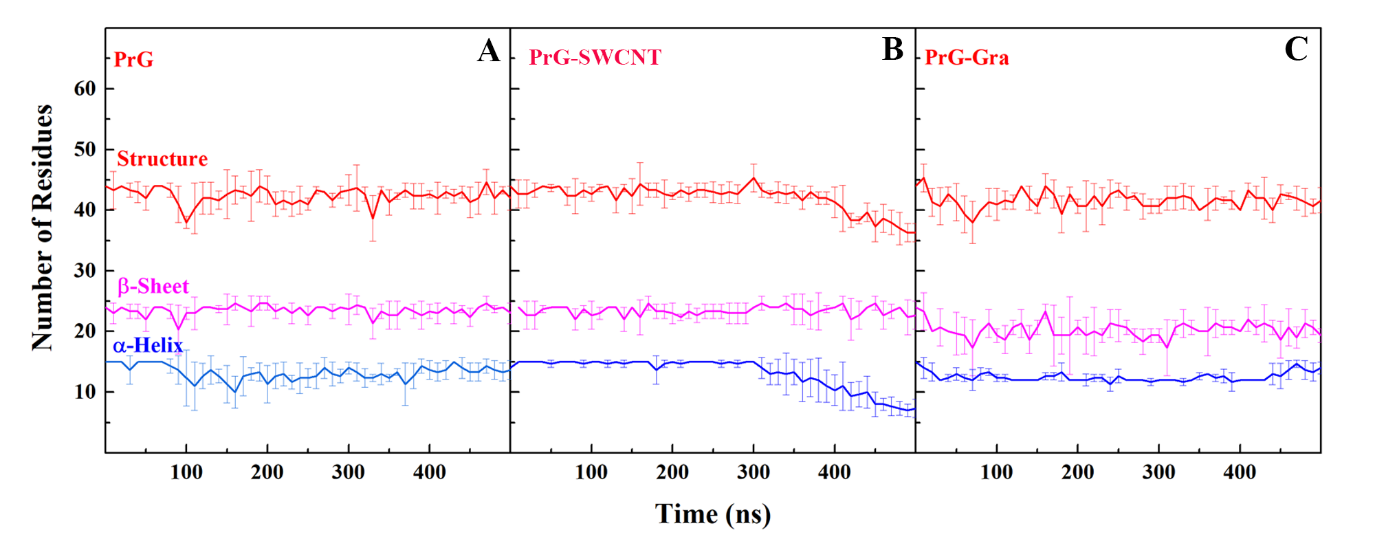


**Figure S5.** Number of residues building the secondary structure in the PrG alone and in presence of SWCNT/Gra during 500 ns of simulation. Analysis of number of the PrG residues forming secondary structure (A) in the PrG alone, (B) in presence of SWCNT, and (C) in presence of Gra.

**Figure S6.**


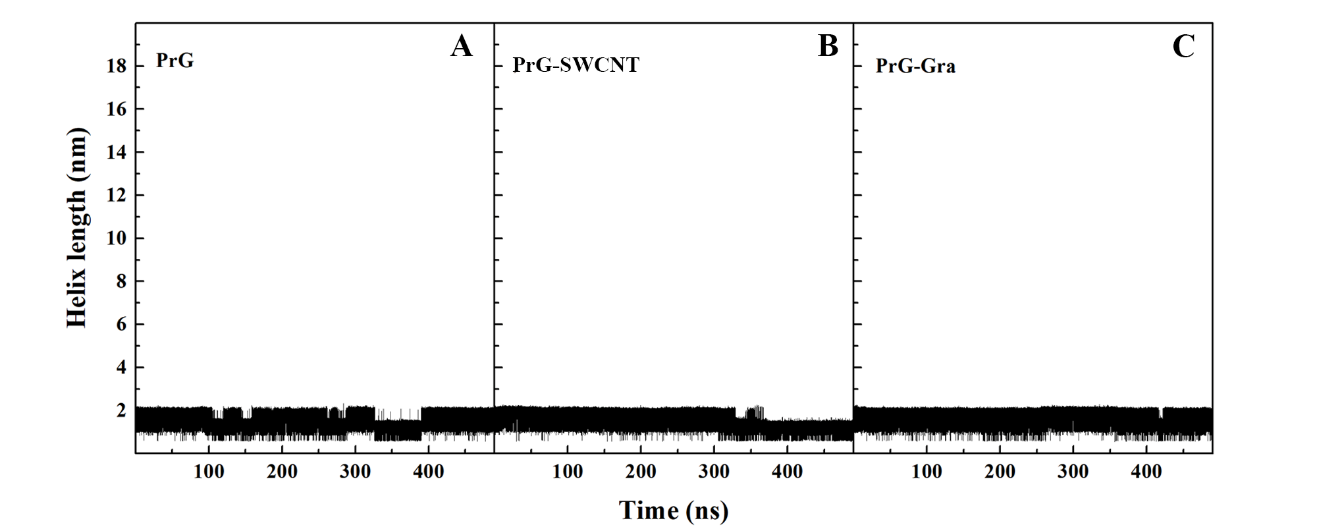


**Figure S6.** Helicity length of the PrG alone and in presence of SWCNT/Gra along 500 ns of simulation study. Helicity length of the PrG (A) alone, (B) in presence of SWCNT, and (C) in presence of Gra.

**Figure S7.**


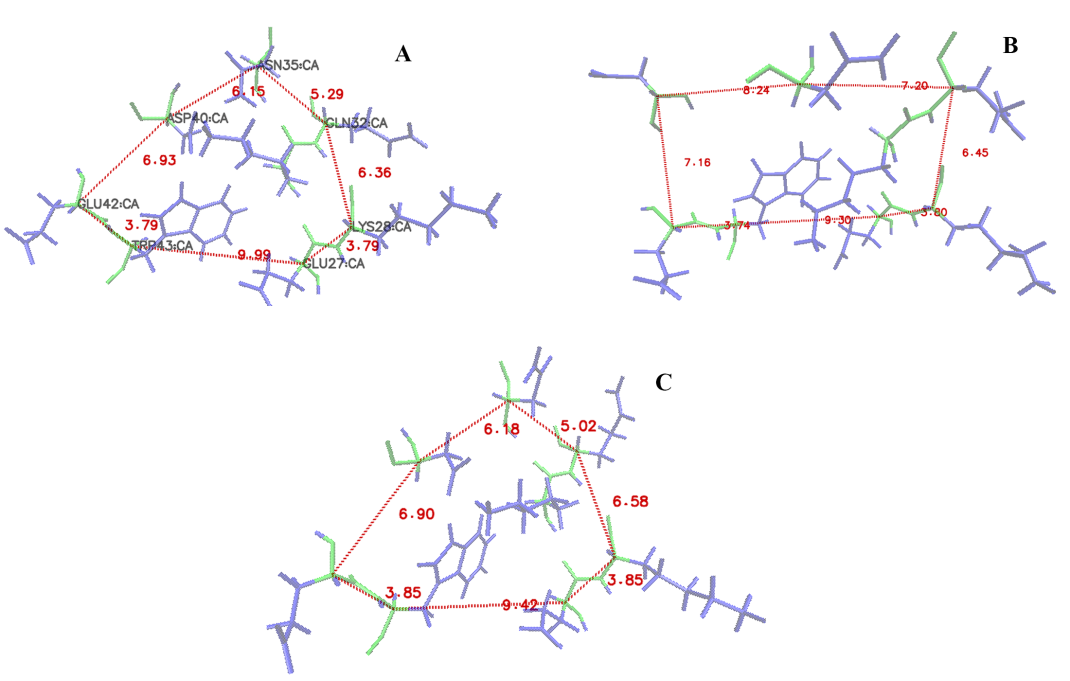


**Figure S7.** The distances between amino acids forming the FcR pocket in (A) the PrG alone, (B) the PrG-SWCNT complex, and (C) the PrG-Gra complex after 500 ns of simulation study.

**Table S1.**

| No | Protein Name | [Carbonaceous](https://www.sciencedirect.com/science/article/pii/S0008622310007207)  NMs | Hydrophobicity index (%) | Number of aromatic residues | Molecular weight (kDa) | pI | [Carbonaceous](https://www.sciencedirect.com/science/article/pii/S0008622310007207)  NMs interaction |
| --- | --- | --- | --- | --- | --- | --- | --- |
| 1 | BSA | SWCNT, MWNT and Graphene | 37.28 | 54 | 66.5 | 5 | Good |
| 2 | Lysozyme | SWCNT and Graphene | 34.88 | 12 | 14 | 9.3 | Very Good |
| 3 | Cytochrome C | SWCNT | 31 | 9 | 11.5 | 9.5 | Very Good |
| 4 | Ferritin (24 domain) | SWCNT | 38.5 | 336 | 470 | 4.6 | Good |
| 5 | Immunoglobulin G | SWCNT and Graphene |  |  | 150 | 7 | Weak |

**Table S1.** Compare some properties of proteins which have significant or insignificant tendency to interact with CNS.

**Table S2.**

| Sample | Alpha helix (%) | Anti-parallel and parallel (%) | Beta-turn (%) | Random- coil (%) |
| --- | --- | --- | --- | --- |
| Native PrG | 26 | 24 | 29 | 21 |
| Sonicated-PrG | 14 | 17 | 32 | 37 |
| PrG-SWCNT | 11 | 27 | 11 | 51 |
| PrG-Gra | 28 | 20 | 27 | 25 |

**Table S2.** Deconvolution analysis has been done with CDNN software to determine the content of PrG secondary structures in different conditions.

**Table S3.**

| No | Protein Name | [Carbonaceous](https://www.sciencedirect.com/science/article/pii/S0008622310007207)  NMs | First secondary structure | Last secondary structure | Methods used for secondary structure analysis |
| --- | --- | --- | --- | --- | --- |
| 1 | BSA | SWCNT | α-helix, β-sheet | β-sheets induced /partially unfolded/not obvious changes observed | CD spectroscopy |
| 2 | Bovine Fibrinogen | SWCNT | α-helix, β-sheet and random coil | β-sheets induced | CD spectroscopy |
| 3 | Immunoglobulin G | SWCNT | α-helix, β-sheet | β-sheets induced | CD spectroscopy |
| 4 | Transferrin | SWCNT | Not induced structural changes | | CD spectroscopy |
| 5 | Fibrinogen | SWCNT | α-helix, β-sheet | β-sheets induced | CD spectroscopy |
| 6 | BSA | Graphene oxide | α-helix, β-sheet | α-helix reduced | CD spectroscopy |
| 7 | hIAPP22–28 | SWCNT and Graphene | β-sheet | β-sheet reduced | MD study |
| 8 | HIV-1 Vpr13-33 | Graphene oxide | α-helix | α-helix reduced | MD study |
| 9 | Lysozyme | SWCNT | α-helix, β-sheet | not obvious changes observed | CD spectroscopy and MD study |

**Table S3.** A summarize of studies focus on secondary structural changes occurred in various types of proteins when bonded to SWCNT or Gra.

**Table S4.**

| Ligand Name | length | M, N | Chirality/termini | diameter | Length of C-C bond | Number of total atoms | Number of layers |
| --- | --- | --- | --- | --- | --- | --- | --- |
| SWCNT | 3nm | 6, 6 | Armchair/nonfunctionalized | 0.81 | 0.1418 | 312 | 1 |
| GRA | Lx=3nm  Ly=3nm | M=N | Armchair/ nonfunctionalized | 4.2 | 0.1418 | 416 | 1 |

**Table S4.** Properties of SWCNT and Gra generated in VMD.
